# The Phenotype of Many Independently Isolated +1 Frameshift Suppressor Mutants Supports a Pivotal Role of the P-Site in Reading Frame Maintenance

**DOI:** 10.1371/journal.pone.0060246

**Published:** 2013-04-04

**Authors:** Gunilla Jäger, Kristina Nilsson, Glenn R. Björk

**Affiliations:** Department of Molecular Biology, Umeå University, Umeå, Sweden; University of British Columbia, Canada

## Abstract

The main features of translation are similar in all organisms on this planet and one important feature of it is the way the ribosome maintain the reading frame. We have earlier characterized several bacterial mutants defective in tRNA maturation and found that some of them correct a +1 frameshift mutation; i.e. such mutants possess an error in reading frame maintenance. Based on the analysis of the frameshifting phenotype of such mutants we proposed a pivotal role of the ribosomal grip of the peptidyl-tRNA to maintain the correct reading frame. To test the model in an unbiased way we first isolated many (467) independent mutants able to correct a +1 frameshift mutation and thereafter tested whether or not their frameshifting phenotypes were consistent with the model. These 467+1 frameshift suppressor mutants had alterations in 16 different loci of which 15 induced a defective tRNA by hypo- or hypermodifications or altering its primary sequence. All these alterations of tRNAs induce a frameshift error in the P-site to correct a +1 frameshift mutation consistent with the proposed model. Modifications next to and 3′ of the anticodon (position 37), like 1-methylguanosine, are important for proper reading frame maintenance due to their interactions with components of the ribosomal P-site. Interestingly, two mutants had a defect in a locus (*rpsI*), which encodes ribosomal protein S9. The C-terminal of this protein contacts position 32–34 of the peptidyl-tRNA and is thus part of the P-site environment. The two *rpsI* mutants had a C-terminal truncated ribosomal protein S9 that destroys its interaction with the peptidyl-tRNA resulting in +1 shift in the reading frame. The isolation and characterization of the S9 mutants gave strong support of our model that the ribosomal grip of the peptidyl-tRNA is pivotal for the reading frame maintenance.

## Introduction

Evolution of the translation apparatus involved in transfer of the genetic message stored in mRNA into proteins was an early event [1]. In the beginning of life translation made many missense errors and it was not possible to translate long mRNAs due to difficulties in maintaining the reading frame. Thus, the evolution of how translation avoids missense and reading frame maintenance errors must have occurred early and before the three domains of life emerged. Therefore, its basic mechanism is most likely similar in all organisms [1]. Many missense errors are not harmful and they occur in cells of to-day at a frequency of about 4×10^−4^ per codon [2] although it varies widely at different sites in bacteria and in yeast [3], [4]. Even if this error level is low, it would still result in that only 78% of the molecules of a 500 amino acid protein having no missense error [5]. Therefore, in a cell many proteins are not faithfully decoded and contain missense errors. Since many of these errors are in non-critical positions of proteins and influence their activity and stability in only minor ways, such an error level is apparently acceptable for the cell. However, every processivity error, such as a frameshift error, is harmful, since ribosomes shifted into the wrong frame will generally soon encounter a stop codon and terminate and thereby generate a truncated peptide. Accordingly, the frequency of processivity error should be lower than missense errors although the estimates of such spontaneous frameshift errors have been difficult to assess [5]. Parker suggested that the frameshift errors may be 10^−5^ or less [2] and thus at least 10-fold less than the level of missense errors.

Although we have learnt much about the mechanism of translation, especially about the 3D structure of the decoding center and how the tRNA is located on the ribosome at the various steps of translation [6]–[8], the mechanism of how the reading frame is maintained is still not known (reviewed in [9]). In some cases there are special sites, the programmed frameshifting sites, at which frameshifting occurs at high frequency due to the presence of various stimulators [10]–[13]. However, at a much lower frequency, frameshift errors may also occur at sites with no apparent nearby stimulators. Such frameshift errors seem to require that the ribosome stalls due to imbalances in any of the steps in the translation elongation process. One way to avoid frameshift errors would be that each step in the elongation cycle occurs at a uniform rate. Indeed, the various aminoacylated tRNA combined with elongation factor Tu (EF-Tu) functions equivalently in translation suggesting that tRNA and its cognate amino acid have co-evolved [14]. Moreover, modified nucleosides, which are present in tRNAs in all organisms, uniform the function of the tRNA [15]. Indeed, mutants isolated as deficient in various modified nucleosides with vastly different chemical structures, present in many different positions of tRNA, and in different tRNA species, induce frameshift errors [16] [17]–[23]. Structural changes of tRNA as well as alterations in elongation factors and rRNA also induce frameshift errors [9], [11]. Moreover, starvation of amino acids, presence of rare or stop codons, or over-expression of tRNA also induce such errors [24]–[27] [28], [29]. Therefore, imbalances in the supply of aminoacylated tRNA and certain sequences in the mRNA upset the maintenance of the reading frame. Accordingly, changes in the environment may also induce errors in reading frame maintenance and indeed, cells in stationary phase have an intrinsic increased rate of frameshift error rate [30], [31].

**Figure 1:**
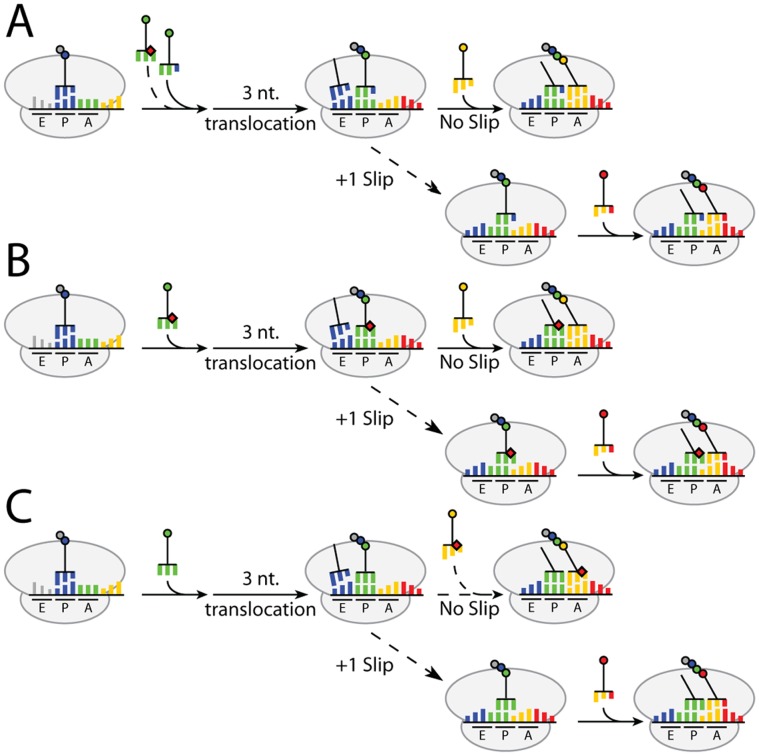
The ribosomal grip of the peptidyl-tRNA is pivotal in reading frame maintenance. The figure shows three ways (A, B and C) how certain events may induce slippage by the peptidyl-tRNA and thereby a frameshift error. It is the ternary complex (aa-tRNA*EfTu*GTP) which enters the A-site and interacts with the codon but in the figure we have symbolized it with “aa-tRNA” to save space. **A**. A defective cognate tRNA (red diamond) is slow (broken arrow) entering the A-site allowing a near-cognate aa-tRNA (blue wobble nucleoside) to decode the A-site codon. After a 3 nucleotide translocation the near-cognate peptidyl-tRNA may slip into the +1 frame. **B**. A defective cognate aa-tRNA (red diamond) decodes efficiently the codon in the A-site. After a 3 nucleotide translocation the defective cognate peptidyl-tRNA may be prone to slip into the +1 frame. **C**. The defective aa-tRNA (red diamond, yellow tRNA) is slow entering the A-site mediating a pause allowing the cognate wild type peptidyl-tRNA to slip into the +1 frame. Not depicted in the figure, alterations in the ribosomal P-site environment may also induce a frameshift error if the alteration changes the ribosomal grip of the peptidyl-tRNA. The figure is adopted from [36] with permission. Indeed, as shown in this paper a truncation of ribosomal protein S9, which interacts with the peptidyl-tRNA induces an error in reading frame maintenance (See Fig. 6). Moreover, the occupancy of the E-site also improves reading frame maintenance [80], [86]–[88], perhaps by strengthening the ribosomal grip of the peptidyl-tRNA. Therefore, a defective tRNA may also increase frameshifting by altering the dissociation rate of it from the E-site.

Transfer RNAs with an extra nucleotide in the anticodon loop suppress certain +1 frameshift mutations [32]. From analysis of such altered tRNAs it was inferred that the frame error induced by an inserted nucleotide in the mRNA was corrected by a tRNA having an apparent four nucleotide anticodon. Such an anticodon was suggested to read four bases, allowing a quadruplet translocation and thereby moving the ribosome into the zero frame [32]. This explanation supported the suggestion that the normal tRNA having a three nucleotide anticodon was used as yardstick in reading frame maintenance by monitoring the three nucleotide translocation required for reading frame maintenance [33]. Although the yardstick model was attractive, it was shown not to be valid for the classical frameshift mutations *sufA6* and *sufB2*, which both have an extra G-nucleotide in the anticodon loop of *proK* 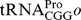 and *proL* 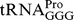, respectively [34]. It was suggested that these tRNAs suppress a +1 frameshift mutation by being defective in A-site entrance and thereby being out-competed by the near-cognate *proM* 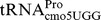. Following a normal three nucleotide translocation, this near-cognate peptidyl-tRNA slips forward one nucleotide thereby moving the A-site codon into the zero frame. In accordance with this model, many base substitutions in the body of *proL* 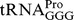, of which the *sufB2* tRNA is a derivative, as well as in the anticodon, also suppress certain +1 frameshift mutations and the frameshift event occurs in the P-site [35]. Based on these observations and how deficiency of many different modified nucleosides imposes a +1 frameshift, an explanatory model was suggested [16], [36]. In the proposed model (Figure 1) the ribosomal grip of the peptidyl-tRNA is a key feature in reading frame maintenance [36]. There are several ways that a defective tRNA can induce frameshifting, *e.g*.**:** Fig. 1, **A**; the ternary complex with the defective tRNA is so slow entering the A-site that it allows a ternary complex containing a near-cognate tRNA to decode the A-site codon. After a normal three nucleotide translocation to the P-site, the peptidyl-near-cognate tRNA is prone to slip into an overlapping reading frame. Fig. 1, **B**; the ternary complex with a defective tRNA decodes the codon in the A-site efficiently, but once the defective tRNA has been translocated into the P-site it may slip on the mRNA. Fig. 1, **C**; the ternary complex containing a defective tRNA is so slow entering the A-site that it causes a pause which allows the wild type peptidyl-tRNA to slip. Note, that various physiological conditions may reduce the level of charged tRNA or the degree of modification and thereby induce a frameshift error according to this model. Basically, as soon as the kinetics of the entry to the A-site and the translocation is not in balance, the ribosome may stall allowing the peptidyl-tRNA to shift frame. This model of frameshifting is similar in many features to other models suggesting P-site slippage [24], [34], [35], [37]–[40] or models proposing that the aberrant peptidyl-tRNA induces a binding of the aa-tRNA in the A-site to the correct frame [39], [41]. In addition we may also expect according to the model that changes in the P-site environment of the ribosome may also induce frameshift errors.

An unbiased test of the proposed model (Fig. 1) would be to first select many independent mutants able to suppress various +1 frameshift mutations and then genetically and biochemically characterize such mutants to see whether or not their frameshift phenotypes are consistent with the model. Here we address this question by isolating and characterizing many (467) independently isolated +1 frameshift suppressor mutants. These 467+1 frameshift suppressor mutants had alterations in 16 different loci. According to the model we expected that many loci would, in some way, influence the structure and the activity of a tRNA. Indeed 15 of these 16 loci were in this class and the mechanism how they induce frameshifts to correct the consequences of the +1 frameshift are all consistent with the model (Figure 1). Interestingly, two mutants had a defect in a loci not altering any tRNA but changing the P-site environment. These two mutants (*rpsI*) had a C-terminal truncated ribosomal protein S9, which C-terminal contacts the peptidyl-tRNA and thereby is part of the ribosomal grip of the peptidyl-tRNA. The isolation and characterization of these *rpsI* mutants gave a strong support of our model that the ribosomal grip of the peptidyl-tRNA is pivotal for the reading frame maintenance.

## Materials and Methods

### Bacteria and Growth Conditions

The bacterial strains used were derivatives of *Salmonella enterica serovar typhimurium* and *Escherichia coli* (Table 1). As rich media Luria-Bertani (LB) was used [42].The minimal solid medium was made from the basal medium [43] with 15g of agar per liter and supplemented with 0.2% glucose and required amino acids and/or vitamins [44]. TYS-agar (10g of Trypticase Peptone, 5g of yeast extract, 5g of NaCl, and 15g of agar per liter) was used as solid rich medium.

**Table 1:** *S. enterica* and *E.coli* strains used in this study.

Strains	Genotype	References
*Salmonella enterica*		
GT853	*hisO1242*, *hisC3737*	Laboratory collection
GT5796	*his-152*, a deletion from the end of *hisD* to the beginning of *hisI*	[83]
GT6315	LT2/pKD46	Laboratory collection
GT6372	(TT17437) pNK2881 (miniTn10 tranposase helper plasmid)/*his-644,serA790,lys-554*	[82]
GT6374	Pool of 35000 colonies with random Tn10dTc insertions in GT6372	This study
GT6402	*proL2231*(G2A), *hisO1242, his C3737, stm2220-2546::*Tn*10d*Tc,	This study
GT6408	*mnmA1*(W38stop), *hisO1242, hisC3737,stm1254-2553::*Tn*10d*Tc	This study
GT6463	*hisC10106*, *zdd-2532::cat*, *hisO1242*	This study
GT6536	*hisC10109*, *hisO1242*	This study
GT6579	pSMP24/*his-152*	This study
GT6588	Pool (170000 colonies) of EZ-R6 gamma KAN, *his-152*, pSMP24)	This study
GT6795- GT6804,GT6806,GT6810	As GT6805 but with *glnU* alleles as shown in Table 4	This study
GT6805	*glnU1538* (G4C), stm0672-2539::Tn10dTc, *hisC3737*, *hisO1242*	This study
GT6807	*hisD10110*, *hisO1242*, *zdd-2532::cat*	This study
GT6828	*hisD10111*, *zdd-2532::cat*, *hisO1242*	This study
GT6995	*proK2236* (G added in AC), *hisD10110, hisO1242*, *zdd-2532::cat*	This study
GT7128	pKD46/*hisD*-2540::tetRA, *zdd2532::cat*, *hisO1242*	This study
GT7170	*mnmA2*(S33F), *hisO1242, hisD10111,stm1221-2545:*:Tn*10d*Tc	This study
GT7279	*proM2219* (G31A), *hisD10111, hisO1242, zdd-2532::cat, stm3930-2547::*MudSacI	This study
GT7318	*iscS56* (S184I), *hisC3737, hisO1242, stm2545-2548::*Tn*10d*Tc	This study
GT7321	*hisD10122*, *zdd-2532::cat*, *hisO1242*	This study
GT7398	*rpsI2* (deletion from R109 to the C-terminal end), *stm3333-2543::*Tn*10d*Tc, *hisD10122*, *zdd-2532::cat*, *hisO1242*	This study
GT7432	*tusE30* (K128stop), *stm1091-2549::*Tn*10d*Tc, hisD10122, zdd-2532::cat	This study
GT7436	*mnmE13* (del aa247-271), *stm3848/3849-2542::*Tn*10d*Tc, *hisD10122, zdd-2532::cat*, *hisO1242*	This study
GT7440	*mnmA3*(G24D), *hisD10122*, *stm1221-2545::*Tn*10d*Tc, *zdd-2532::cat*, *hisO1242*	This study
GT7453	*tusB27, stm3453-2550::*Tn*10d*Tc, *hisD10122*, zdd-2532::cat, *hisO1242*	This study
GT7458	*hisT10128* (deletion), stm2375-2552::Tn10dTc *hisD10122*, zdd-2532::cat, *hisO1242*	This study
GT7478	*mnmG19* (*gidA*, 58 bp deletion from A402), *stm3848/3849-2542::*Tn*10d*Tc, *hisD10122*, zdd-2532::cat, *hisO1242*	This study
GT7484	*ybbB219 (*G67R), *sfbA-2537::*MudSacI *hisD10122*, *zdd-2532::cat*, *hisO1242*	This study
GT7644	Deletion between *tusD* (A35) and *tusC* (S41), *hisD10111, stm3453-2554::*Tn*10d*Tc, *hisO1242*	This study
GT7690	*trmD39* (D150G), *hisO1242*, *hisD10111*, *zdd-2532::cat*	This study
GT7747	*rpsI3* (deletion from E99 to the C-terminal end), *stm3346-2551::*Tn*10d*Tc, *hisD10122*, *zdd-2532::cat*, *hisO1242*	This study
GT7775	*proL2240, stm2235-2541*::Tn*10d*Tc, *hisD10122, hisO1242, zdd*-2532::cat	This study
GT7829	*mnmG20* (*gidA*, G340stop) *pstA2544::*Tn*10d*Tc, *hisD10122*, *zdd-2532::cat*, *hisO1242*	This study
GT7901	*tusA32* (C19Y), *hisD10122, hisO1242*	This study
GT7988	*proL2241* (G10A), *hisO1242*, *hisD10111*, *zdd-2532::cat, zej-2555::*Tn*10d*Tc	This study
Escherichia coli		
DH5alfa	*fhuA2* Δ*(argF-lacZ)U169 phoA glnV44 Φ80* Δ*(lacZ)M15 gyrA96 recA1 relA1 endA1 thi-1 hsdR17*	Laboratory collection
pNTR-SD-*trmD*	*trmD* under IPTG-inducible promoter	[54]
pNTR-SD-*mnmA*	*mnmA* under IPTG-inducible promoter	[54]
pNTR-SD-*iscS*	*iscS* under IPTG-inducible promoter	[54]

### Genetic Procedures

Transduction with phage P22 HT105/1 (*int-201*) [45] was performed as previously described [44]. DNA sequencing was performed on chromosomal DNA or PCR products following the manual of Applied Biosystems ABI PRISM Cycle Sequencing Ready Reaction Kit Big Dye™.

### Systems Used to Isolate +1 Frameshift Suppressor Mutants

We have used different +1 frameshift mutations in the *hisC* or the *hisD* genes (Table 2) constructed as described below. The *hisC3737* mutation was used earlier to obtain several of the classical frameshift suppressor mutants [46] and apparently only very small amount of the HisC enzyme is required to enable a mutant to form colonies within a day or two without histidine in the growth medium. We also introduced other frameshift mutations at the same site as in *hisC3737* to widen our possibilities to obtain various frameshift suppressor mutants. Several frameshift mutations in the *hisD* gene were constructed, since only 1% of the HisD enzyme is enough to make a cell His^+^ within a day [47]. Thus, only a fraction of a percent suppression of a frameshift mutation in the *hisD* gene is required to make enough of a functional HisD enzyme to allow a colony to appear within a few days. Monitoring suppression of a frameshift mutation in the *hisD* gene would be a sensitive way to detect mutations mediating very weak suppressor activity. Indeed, monitoring frameshifting as growth on a plate lacking histidine is a more sensitive way to monitor +1 frameshift suppression than monitor the suppression of the same +1 frameshift mutation in the *lacZ* gene which encodes β-galactosidase [36].

**Table 2:** Sequence of the various frameshifts sites in the *his*-operon used in the selection of 460 independent frameshift suppressor mutants.

Allele number	Sequence of the frameshift window
*hisC3737*	AUG-(NNN)_124_-GTA-GAG-(NNN)_31_-**CCC-CAA**-UAA
*hisC10106*	AUG-(NNN)_124_-GTA-GAG-(NNN)_31_-**CCC-AUG-** UAA
*hisC10109*	AUG-(NNN)_124_-GTA-GAG-(NNN)_31_-**CCC-UGG-** UAA
*hisD10110*	AUG-(NNN)_4_-CUG-AUU-GAC-UGG-AAC-AGC-UGU-**CCC-UAU**-UGA-ACA-GCA-G
*hisD10111*	AUG-(NNN)_4_-CUG-AUU-GAC-UGG-AAC-AGC-UGU-**CCC-AAG**-UGA-ACA-GCA-G
*hisD10122*	AUG-(NNN)_4_-CUG-AUU-GAC-UGG-AAC-AGC-UGU-**CCC-CAA**-UGA-ACA-GCA-G

1. The sequences of (NNN)_124_ and (NNN)_31_ in *hisC3737*, *C10106* and *C10107* are: agc act gaa aac act ctc agc gtc gct gac tta gcc cgt gaa aat gtc cgc aac ctg gta ccg tat cag tct gcc cgc cgt ctg ggc ggt aac ggc gat gtc tgg ctg aac gcg aat gaa ttc ccg aca gcg gtg gag ttt cag ctc acc caa caa acg ctt aac cgc tac ccg gaa tgc cag cca aag gcc gtg att gaa aac tac gcg caa tat gct ggc gta aag ccg gag cag gtg ctg gtc agc cgc ggc gcg gat gaa ggg atc gag ctg gtg atc cgc gcc ttc tgt gaa ccg ggg aaa gac gcc att ctc tac tgt ccg ccc act tac ggt atg tac agc gtc agc gcc gaa acc att ggc and: g*ta g*ag cgc cgg acg gtt ccc gcg ctt gaa aac tgg cag ctg gat cta cag ggg att tcc gac aac ctt gac ggc aca aaa gtg gtg ttc gtt tgt agc ccc caa *taa* [84], respectively.

2. The sequence of (NNN)_4_ in *hisD10110*, *hisD10111* and *hisD10122* is: AGC-UUC-AAU-ACC.

### Constructions of Various +1 Frameshift Mutations in the *hisC* and *hisD* Genes

The frameshift mutation *hisD10122* was constructed as follows: The tetracycline resistance genes *tetA* and *tetR* from Tn*10d*Tc were first inserted into the *hisD^+^* gene in strain GT6808 (*zdd-2532::cat, hisO1242*) generating strain GT7127 (*hisD10132::tetRA, zdd-2532::cat, hisO1242*) to which plasmid pKD46 from strain GT6315 (LT2, pKD46) was introduced resulting in strain GT7128 (pKD46/*hisD10132*::*tetRA*, *zdd-2532*::*cat*, *hisO1242*). This latter strain was transformed with a 60 nt DNA oligonucleotide designed to replace the tetracycline resistance cassette with the designed frameshift mutation selecting tetracycline-sensitive recombinants [48]; e.g. to construct the frameshifting site CCC-CAA-U present in *hisD10122* mutant the codons 13–14 (AGC-CCU) of *hisD* were replaced by CCC-CAA-U. In order to produce a functional HisD protein from the mRNA of this mutant, the ribosome has to shift to the +1 reading frame before the UGA (stop) codon, which is in the zero frame and placed just after the CCC-CAA sequence. In *hisD10122* a +1 shift occurs when 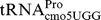 is in the ribosomal P-site at the CCC codon resulting in a mutant peptide sequence (-Cys-Pro-Asn-Glu-). The *hisD10110* and *hisD10111* were constructed similarly by replacing the codons 13–14 (AGC-CCU) of *hisD* with the codons CCC-UAU-U and CCC-AAG-U, respectively, using 60 bp oligonucleotides with the mutated codons in the middle of the oligonucleotides. The complete frameshift windows used in this study are listed in Table 2.

The *hisC3737* mutation is 31codons from the start codon and it creates a CCC-CAA- sequence upstream of the stop codon UAA in the zero frame (See Table 2). The sequence CCC-CAA of *hisC3737* was replaced by CCC-AUG- and CCC-UGG in *hisC10106* and *hisC10109*, respectively, by using the suicide plasmid pDM4 as described [49]. Two complementary 20-mers containing the desired mutation and two primers just outside the *hisC* gene, were used to generate by crossover PCR a fragment that was cloned into a T/A overhang vector. Then the entire *hisC* fragment with the mutation was cut out and ligated into the suicide vector pDM4, which contains the *sacB* gene, and cannot survive on plates with 5% sucrose. The construct was conjugated into *S. enterica* and a derivative, in which the integrated suicide vector had recombined out of the chromosome, were selected by adding 5% sucrose to the agar plates. Transconjugants surviving on plates with 5% sucrose were chosen and correct frame shift construction was confirmed by PCR and sequencing.

### Mutagenesis to Obtain +1 Frameshift Suppressor Mutants

Mutagenesis of the strain GT6588 [pool of EZ-R6 gamma KAN, *his-152* (deletion of the *his*-operon), pSMP24], which contains plasmid pSMP24 harboring *dinB*, was performed by inducing the expression of DinB [50], [51] from plasmid pSMP24 [52]. An over-night culture of strain GT6588 was diluted 2×10^6^-fold and inoculated into 500μl of LB+100 µg carbenicillin (Cb)/ml +0.08% L-arabinose. After 24 hours of growth at 37**°**C, phage P22 was added to make phage lysates. The phage lysates were used to infect strains containing the different frame shifting sites and His^+^ clones were collected every day for nine days and saved for further analysis.

To mutagenize with hydroxylamine a phage P22 lysate prepared from strain GT6374 [pool of random Tn*10d*Tc insertions in strain GT6372 (containing a deletion of the *hisD* and *hisC* genes (*his-644*), thus avoiding recombination with wild type *his*-operon in transductions)] was treated with hydroxylamine as described [53] until approximately 0.1 per cent infectious phage P22 particles remained. This lysate was used to transduce strains with a +1 frameshift mutation as described in Table 2.

Nitrosoguanidine (NG) induced mutations were obtained by placing a crystal of NG on a lawn of the donor strain with either a deletion of the *his*-operon or with the same +1 frameshift mutation as the recipient strain; e.g. *hisD10122* on an agar plate, which was incubated over night at 37**°**C. Around the NG crystal a ring of growing bacteria emerged that contains bacteria with mutations induced by NG. Bacteria were scraped off from several fractions of the bacterial ring and resuspended in 1 ml of LB and allowed to grow for 1–2 hours before phage P22 was added to make lysates. As above the phage lysate was used to infect His^-^ strain harboring a suitable +1 frameshift mutation in the *his*-operon and His^+^ clones were collected as above. To avoid siblings only one mutant from each phage stock was saved. To locate the extragenic +1 suppressor mutation in each His^+^ clone, a Tn*10d*Tet was placed close to the mutation by using a phage P22 stock grown on a random pool of Tn*10d*Tet in strain GT6374 as donor and the His^+^ clone as recipient. Tet^R^ clones were selected and His^-^ clones were screened. These clones have most likely the +1 frameshift mutation exchanged by the wild type allele from the donor. Following verification that the Tn*10d*Tet was close to the +1 frameshift suppressor mutation, the location of the Tn*10d*Tet was determined by DNA sequencing directly on purified chromosome and with primers binding in the transposon and pointing outwards. The chromosome was purified by Qiagen Tip100/G as described by the manufacturer.

### Complementation Analysis

A set of mobile plasmids containing most of the ORFs from *E. coli* with the expression controlled by P_tac_/lacI^q^, was a kind gift from National Institute of Genetics, Mishima, Shizuoka 411–8540, Japan [54]. A plasmid, which contained the wild type copy of the structural gene for the potential mutated gene in the +1 frameshift suppressor mutant, was introduced to this mutant. The frameshift suppressor phenotype was scored as growth on plates lacking His and in several cases the modification of tRNA was established by HPLC. If the plasmid reversed the suppressor phenotype to the wild type phenotype (i. e from His^+^ to His^-^), the mutant was defective in the gene harbored on the plasmid. This was verified by determining the DNA sequence of the mutated gene on the chromosome.

### Analysis of tRNA Levels

Strains were grown in 10 ml LB medium at 37°C to about 4×10^8^ cells/ml. Following centrifugation the pellet was suspended in 1 ml of ice-cold water. Cells were collected by centrifugation and resuspended in 400 µl of 10 mM Tris-EDTA, pH 7.5. The same amount of acid phenol was added and the mixture was vigorously shaken for 10 sec, incubated for 45 min at 65°C with occasional shaking before the phases were separated by centrifugation. To the water phase 400 µl chloroform was added and the mixture was shaken after which the water phase was transferred to a clean test tube. The RNA was precipitated by adding 40 µl of 3M sodium acetate, pH 5.3 and 1 ml of 100% ethanol. The precipitated RNA was washed once with 70% ethanol, centrifuged, and dissolved in 50 µl of water. About 5 µg of RNA was applied to 8% polyacrylamide gel containing 8M Urea in 89 mM Tris-borate buffer pH 8.2 containing 2 mM EDTA. The gel was transferred to a Zeta probe membrane and the RNA was UV cross-linked to the membrane. The tRNAs were detected by Northern hybridization using radioactive oligonucleotides complementary to tRNA^Arg^ or 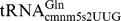 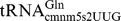.

### Determination of Aminoacylation of 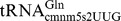 in vivo

Cells were grown in 30 ml LB medium at 37°C to about 4×10^8^ cells/ml and cells were collected by centrifugation. Cells were resuspended in 1 ml of water, washed once with 1 ml of water, and finally resuspended in 500 µl of cold 0.1 M NaAc (pH 4–5) containing 10 mM EDTA. To the suspension of cells, 200 µl of glass beads and 500 µl of 25:24:1 phenol-chloroform-isoamylalcohol mixture was added and the mixture was vortexed four times for one minute with a one minute on ice between the shakings. Following centrifugation, the supernatant was transferred to a new tube and RNA was precipitated by adding 3 volumes of ethanol. The RNA was dissolved in 50 µl of 10 mM NaAc pH 4.5 containing 1 mM EDTA. Half of the sample was diluted with equal volume of 0.5M Tris HCl, pH 9.0 for 20 min at 37°C. The deacylated and non-treated samples were run on an acidic gel containing 8% polyacrylamide, 8 M urea, 0.1 M NaAc pH 5.0. RNA was transferred to Zeta probe membrane and 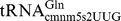 was detected as above.

### Analysis of Modified Nucleosides in tRNA

Bacterial strains were grown over night in LB medium, diluted 100 times in 100 ml of the same medium and grown at 37**°**C to 100 Klett units (approximately 4×10^8^ cells/ml). Cells were lysed and total RNA was prepared [55] and dissolved in 2 ml buffer R200 (10 mM Tris-H_3_PO_4_, pH 6.3, 15% ethanol, 200 mM KCl) and applied to a Nucleobond® AX500 column (Macherey-Nagel Gmbh & Co., Düren, Germany), pre-equilibrated with the same buffer. The column was washed once with 6 ml R200 and once with 2.5 ml R650 (same composition as R200, except for 650 mM KCl instead of 200 mM KCl). Finally, tRNA was eluted with 7 ml R650, precipitated by 0.7 volumes isopropanol, washed twice with 70% and dissolved in water. tRNA was digested to nucleosides by nuclease P1 followed by treatment with bacterial alkaline phosphatase at pH 8.3 [56]. The hydrolysate was analyzed as described earlier [57] using a Supelcosil C-18 column (Supelco) with a Waters Alliance HPLC system.

### Determination of the Amino Acid Sequence of the Slippage Junction

To monitor ribosomal slippage and to purify the slippage product, a previously described system was used [58], [59]. It employs a fusion protein consisting of maltose-binding protein (MBP) fused to glutathione-S-transferase (GST) at its N-terminus and having six histidine residues (6×His) at its carboxy terminus (GST-MBP-6×His). The full-length GST-MBP-His_6_ fusion proteins were expressed from plasmid pUST290 (CCC-CAA-), pUST292 (UUU-CAA), pUST310 (CCC-CAA) or pUST311 (CCC-AAG). These plasmids were constructed by cloning a DNA fragment containing the frameshift sequence into the *BamHI* and *EcoRI* sites of vector pGHM57 [58]. Ligated plasmids were transformed into strain DH5α, analyzed by sequencing the insert, and retransformed by electroporation into different *S. enterica* strains. The fused GST-MBP-6xHis protein expressed from these plasmids were purified essentially as described by Atkins *et al* [58], except the Ni-NTA purification was omitted and the MBP-His_6_ part of the fusion was released by digestion with PreScission Protease (GE Healthcare), while the GST part was still bound to Glutathione-Sepharose. The MBP-His_6_ peptides were separated by SDS polyacrylamide (15%) gel electrophoresis and electroblotted to a Sequi-Blot PVDF membrane (Bio-Rad). The bands corresponding to the MBP-His_6_ peptides were excised from the membrane and subjected to N-terminal sequence analysis by Edman degradation. A +1 reading frame shift when tRNA^Pro^ is in the P-site at the CCC codon in pUST310 would result in the sequence GPLGILI**C**PNDK. Unmodified cysteine is too reactive during N-terminal sequencing and is usually only seen indirectly as the absence of an amino acid in one cycle. The confirmation of the presence of cysteine has been described earlier [36].

### Nomenclatural Acts

The electronic edition of this article conforms to the requirements of the amended International Code of Zoological Nomenclature, and hence the new names contained herein are available under that Code from the electronic edition of this article. This published work and the nomenclatural acts it contains have been registered in ZooBank, the online registration system for the ICZN. The ZooBank LSIDs (Life Science Identifiers) can be resolved and the associated information viewed through any standard web browser by appending the LSID to the prefix “http://zoobank.org/”. The LSID for this publication is: urn:lsid:zoobank.org:pub: XXXXXXX. The electronic edition of this work was published in a journal with an ISSN, and has been archived and is available from the following digital repositories: PubMed Central, LOCKSS [author to insert any additional repositories].

## Results

### 1. Systems and Procedures Used to Isolate Many Independent +1 Frameshift Suppressor Mutants

According to our frameshift model [16], [36] the shift in frame occurs not by an error in the A-site as *e.g.* suggested by the quadruplet translocation model, but by an error in the P-site: *i.e* it is the peptidyl-tRNA that slips forward one nucleotide resulting in a +1 frameshift (Fig. 1). There are several ways that may induce a shift in frame (see Introduction). To extensively test the model in an unbiased way, we here present the characterization of many independently isolated mutants able to suppress various +1 frameshift mutations. These were, after the initial selection, subjected to a careful analysis of their frameshift suppressing phenotype. Such an analysis would reveal whether or not their frameshifting phenotypes were consistent with the model.

We have used different +1 frameshift mutations in the *hisC* and *hisD* genes (Table 2) to isolate extragenic +1 frameshift suppressors. As described in the Materials and Methods, monitoring frameshifting as growth on a plate lacking histidine is a very sensitive way to isolate weak +1 frameshift suppressor mutants allowing us to isolate a wide range of different +1 frameshift suppressor mutants and thereby extensively test our frameshift model. Furthermore, we placed the frameshift mutations (both the *hisC* and *hisD* derivatives) on the chromosome (not on a plasmid!) ensuring a “wild type balance” of various factors involved in reading frame maintenance. We feel this is important since overexpression of mRNA, as is the case if the test gene containing the frameshift mutation is residing on a plasmid, or unbalanced tRNA pools may induce translation errors [[60], [61]discussed by Atkins and Björk [9]]. Therefore, we expected these systems to be good tools to extensively test our model.

To isolate many independent frameshift suppressor mutants, a strain having a partial deletion of the *his*-operon (*his-152*) was mutagenized by overproduction from the plasmid pSMP24 (*dinB^+^*) of DinB, which induces random mutations of various kinds [50]. Alternatively, we mutagenized cultures of strains harboring one of the indicated *his*-mutations or a *his* deletion by nitrosoguanine (NG). Phage P22 were grown on such cultures and used to infect strains having one of the *his*-mutations shown in Table 2. His^+^ transductants were selected at 37°C following several days of incubation to allow the appearance of weak suppressor mutants. Care was taken to avoid siblings by saving only one unique mutant from each phage stock. Thus, all mutants characterized (Table 3) are of independent origin even if the mutation resulted in the same nucleotide substitution. Next, we placed a Tn*10d*Tc transposon close to each +1 frameshift suppressor mutation by crossing out the suppressor phenotype (the His^+^ phenotype of the suppressor mutant changed to the parent phenotype His^-^ when the transposon is located close to the suppressor mutation). The location of the transposon on the chromosome was determined by DNA sequencing out from the transposon and into the nearby chromosomal region. To link the His^+^ phenotype with the mutated gene, we transduced it back to the parental strain (His^-^) by selecting Tet^R^. Frequency of co-transduction between the transposon and the suppressor phenotype (His^+^) indicated which gene mediated the +1 frameshift suppressor phenotype. If DNA sequencing showed that the suspected gene contained a mutation, we introduced a plasmid harboring a wild type copy of the mutated gene to complement the mutation and thereby further demonstrate the link between the mutated gene and the +1 frameshift suppressor ability. In this way we obtained and characterized 467 independent mutants harboring an extragenic suppressor to the different +1 frameshift mutations in the *his*-operon. These 467+1 frameshift suppressor mutations present in the mutants were distributed in 16 different loci (Table 3).

**Table 3:** Summary of all mutants selected as suppressors to various frameshift mutations in the *his*-operon^a^.

Gene mutated	His mutationused	No of mutants obtained	Molecules affected	Comments	Mechanism of frameshifting (See Fig. 1)
*mnmA*	*D10122*	39 (24^b^)	Synthesis of mnm^5^s^2^U34	Induces s^2^-deficiency of mnm^5^s^2^U34	Alt. **C** in Fig. 1
*----« ----*	*C3737*	1	----« ----	----« ----	----« ----
*----« ----*	*D10111*	1	----« ----	----« ----	----« ----
*tusA*	*D10122*	2	----« ----	----« ----	----« ----
*tusDCB*	*D10122*	15	----« ----	–– « –----	----« ----
*----« ----*	*D10111*	1	––« ----	––« ––	----« ----
*tusE*	*D10122*	1	----« ----	----« ----	----« ----
*iscS*	*C3737*	7	----« ----	----« ----	----« ----
*mnmE*	*D10122*	2	----« ----	Induces lack of mnm^5^-sidechain of mnm^5^s^2^U34	----« ----
*gidA*	*D10122*	1	----« ----	----« ----	----« ----
*–« –*	*D10111*	2	----« ----	----« ----	----« ----
*ybbB*	*D10122*	1	Adds a geranyl group to the s^2^-group of mnm^5^s^2^U34	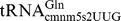 Results in decreased level of charged	----« ----
*hisT*	*D10122*	10 (7^b^)	Synthesis of Ψ at postion 38, 39 and 40 i some tRNAs	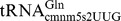 Induces lack of Ψ in *e.g.*	----« ----
*glnU*	*D10122*	93 (82^b^)	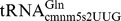 Alters thestructure of	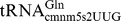 Induces reduced level of charged	----« ----
*----« ----*	*C3737*	13	----« ----	----« ----	----« ----
*glnS*	*D10122*	1	Alters the structure of GlnRS	––« ––	----« ----
*proL*	*D10122*	85^b^	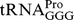 Alters the structure of or delete	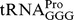 Induces deficiencyof Pro-	Alt **A** in Fig. 1
*----« ----*	*C3737*	21 (3^b^)	----« ----	----« ----	----« ----
*----« ----*	*C10106*	57	----« ----	----« ----	----« ----
*----« ----*	*D10110*	9	----« ----	----« ----	----« ----
*----« ----*	*D10111*	38^b^	----« ----	----« ----	----« ----
	*C10109*	43			----« ----
*proM*	*D10111*	1	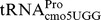	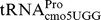 Induces a structural alteration of	Alt **B** in Fig. 1
*proK*	*D10110*	2	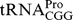	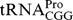 Induces structural alterations in	Alt **A** and **B** in Fig. 1
*trmD*	*C3737*	2	Alters the tRNA(m^1^G37) methyltransferase	Results in m^1^G37 deficiency in e.g. all three proline tRNAs	Alt **B** in Fig. 1
*----« ----*	*D10110*	1	----« ----	----« ----	----« ----
*----« ----*	*D10111*	16 (13^b^)	----« ----	----« ----	----« ----
*rpsI*	*D10122*	2	Ribosomal protein S9	Results in C-terminal truncated version of ribosomal protein S9	**Changed P-site environment** (See Fig. 6.)
		Total no. of mutants : **467**			

a)The various *his*-alleles used were: *hisD3737* (CCC-CAA), *hisC10106* (CCC-AUG), *hisC10109* (CCC-UGG), *hisD10110* (CCC-UAU), *hisD10111* (CCC-AAG), or *hisD10122* (CCC-CAA).

b)These mutations were not sequenced but localized by transduction to a transposon closely linked to the indicated gene. The linkage between the transposon and the mutation was consistent with the mutation being in the indicated gene.

### 2. Alterations of the Primary Sequence of 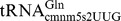 (106 mutants) or an Alteration of the Gln-tRNA Synthetase (One Mutant) Induce Low Concentration of Charged Gln-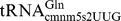, which causes Slow Entry into the A-site and thereby Allowing a +1 Frameshift in the P-site

A glutamine codon is present in two of the six frameshift sites used (Table 2). We therefore expected mutations influencing the activity of 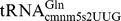 and indeed this was the case.

**Figure 2:**
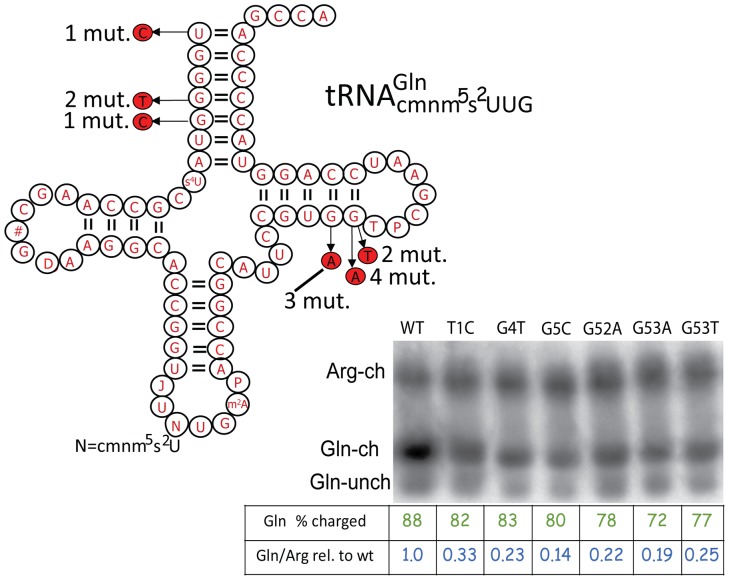
Sequence and aminoacylation levels *in vivo* of various mutant 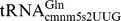:s. The positions of charged (ch) Gln or Arg-tRNA and uncharged (unch) Gln-tRNA are indicated and their migration pattern was obtained from control experiments. The position of uncharged Arg-tRNA is between Gln-ch and Arg-ch as shown by a control experiment. Since Arg-tRNA was 100% charged the uncharged Arg-tRNA is not indicated in the figure. Sequence of wild type *glnU* 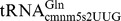 and various mutants (base alteration shown in red). s^4^U, 4-thiouridine, #, 2′-O-methylguanosine (Gm), D, dihydrouridine, J, 2′-O-methyluridine (Um), N, 5.-carboxymethylaminomethyl-2-thiouridine (cmnm^5^s^2^U), m2A, 2-methyladenosine, P, pseudouridine (Ψ), T, 5-methyluridine (m^5^U).

We obtained 93 mutants as extragenic suppressors in the *glnU* gene to the *hisD10122* mutation based on their linkage to a transposon close to the structural gene (*glnU*) for 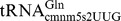. Of these 11 were verified by determination of the *glnU* sequence. We also obtained 13 mutants as extragenic suppressors to *hisC3737*, which were sequenced and further analyzed (Table 4, Fig. 2). Three of these mutants displayed a temperature sensitive phenotype (*glnU1529-30, 1538*), nine (*glnU1526-28; 1531-33;1535-37*) were cold sensitive, and one mutant (*glnU1537*) had a reduced growth which was similar at all three temperatures tested (Table 4). According to our model (Fig. 1), we suspected that alterations of 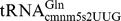 or a defect of Glu-tRNA synthetase should reduce the level of charged Gln-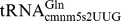. Transfer RNA was prepared from wild type and from the various mutants under conditions which preserve the aminoacylation of tRNA [62]. Although there was no difference in the relative level of glutaminyl-tRNA in the mutants compared to tRNA from the wild type (Fig. 2), a reduced level of 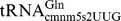 relative to tRNA^Arg^ was observed in the mutants. We conclude that the alterations in all *glnU* mutants did not affect the charging of 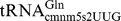, but made the tRNA more unstable resulting in a lower concentration of the Gln-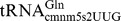 than in the wild type mediating +1 frameshifting consistent with our model.

**Table 4:** Growth and suppressor ability of mutants with structural changes in 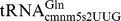.

Mutants(allele number)	No. of indep.*glnU* mut.	Mutations	Efficiency of suppression^a)^(E+glu/E+glu+His)	TYS^b)^ 30 °C(2 days)	TYS^b)^ 37 °C(1 day)	TYS^b)^ 42.5 °C(1 day)	Growth Phenotype^c)^
1537	1	T1C	**0.2**	**0.8**	**0.7**	**0.8**	**Similar reduction at all temp.**
1529, 1530	2	G4T	**0.1**	**0.9**	**1.1**	**0.7**	**Ts**
1538	1	G5C	**0.6**	**0.8**	**0.7**	**0.5**	**Ts**
1526-1528	3	G52A	**0.7-1.1**	**0.3r**	**0.4**	**0.6**	**Cs**
1533, 1535-1537	4	G53A	**0.3**	**0.6**	**0.6**	**0.9**	**Cs**
1531-1532	2	G53T	**0.3**	**0.6**	**0.7**	**0.9**	**Cs**
WT			0	**1.0**	**1.0**	**1.0**	

a)Figures represent the relative colony size of the mutant on minimal glucose plates without histidine after 3 days of incubation at 37°C to the colony size on plates with histidine at the same temperature. Thus, this measurement is an estimation of suppression efficiency corrected for the general growth reduction of the mutant. Ts, temperature sensitive growth phenotype and Cs, cold sensitive growth phenotype.

b)Figures represent colony size relative to the size of wild type control on rich plates (TYS) at indicated temperatures and time of incubation. The colony size of the wild type was 3.2, 2.2, and 2.7 mm at 30, 37 and 42,5°C, respectively.

c) Cs, Cold sensitive; Ts, temperature sensitive.

**Figure 3:**
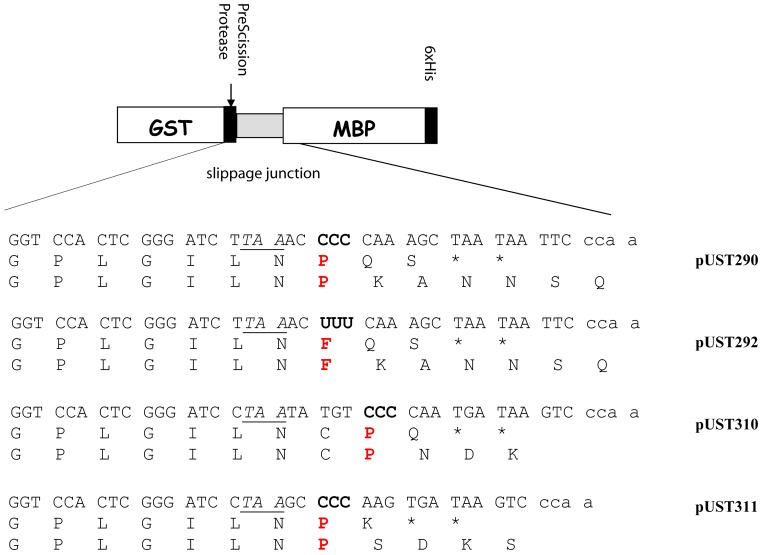
Amino acid sequence of the frameshift product encoded from plasmids pUST290, pUST292, pUST310, and pUST311. The frameshift window, within which the frameshift must occur, is bordered by the stop codon UAA (italics and underlined) in +1 frame and the stop codon UGA UAA in the zero frame (Indicated by a * below the DNA sequence). P or F (in red) denote the last amino acid decoded in the zero frame found in the frameshift product.

Two mutants, *glnU1538* (G5C) and *glnU1531* (G53T) were chosen to determine the frameshift event at the site of the *hisC3737* mutation. For that purpose the plasmid pUST290 was constructed (Fig. 3). The frameshift sequence was inserted between the genes encoding glutathione-S-transferase (GST) and maltose-binding protein (MBP, encoded by the *malE* gene) with six histidine residues (6×His) at the carboxy terminus in the *gst-malE* fusion gene. *malE* is in the +1 frame relative to *gst*, explaining why the complete GST-MBP-6×His fusion protein is only synthesized when a +1 frameshift occurs. If +1 frameshifting does not occur, translation terminates at the UAA stop codon present downstream of the *gst* gene and only GST is produced (Fig. 3). The complete fusion protein was purified from strains containing plasmid pUST290 and *glnU1531* or *glnU1538* mutations using the GST and 6×His affinity tags. To liberate the slippage junction fused to the MBP-6×His, the frameshift product was treated with PreScission Protease. This protease cuts the protein at the specific protease site between the GST moiety and the rest of the peptide. The N-terminus of the slippage junction fused to MBP-6×His was sequenced. From both mutants the first 15 amino acids of the peptide were determined as GPLGILN**P**-KANNSQL, where P (proline) was the last amino acid inserted in 0 frame suggesting that the frameshifting tRNA at the frameshift site CCC-CAA-UGA in *hisC3737* was a wild type pro-tRNA and not the altered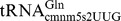.

In order to analyze the influence the P-site tRNA exerts on the frameshifting event we also constructed a plasmid pUST292 with UUU (Phe) instead of the CCC (Pro) codon (Fig. 3). The sequence of this frameshift peptide revealed that it was the wild type 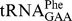 in the P-site that caused the frameshifting event (Fig. 3). Thus, in both cases low concentration of the Gln-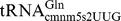 caused the wild type peptidyl-tRNA (Pro or Phe) to slip forward one nucleotide and thereby moving the ribosome into the zero frame. Since both wild type Pro- and Phe-peptidyl tRNA, which interact with two different codons in the P-site were induced to slip by a ribosomal pause, the identity of the last amino acid in the peptiyl-tRNA and the anticodon-codon interaction in the P-site is not critical. Thus, the frameshift phenotype of these mutants was consistent with our model (Fig. 1, alt. C).

Reduced charging capacity may also occur if the Gln-tRNA synthetase (GlnS) is defective. Indeed we obtained a GlnS mutant with an alteration (N70S) changing the environment where the CCA-end of 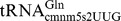 binds to GlnS during the glutaminylation reaction [63]. Since this is an essential gene it was not surprising that we only obtained one mutant.

### 3. Deficiency of the Wobble Nucleoside cmnm^5^s^2^U in 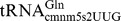 causes a +1 Frameshift in the P-site (72 Independently Isolated Mutants)

**Figure 4:**
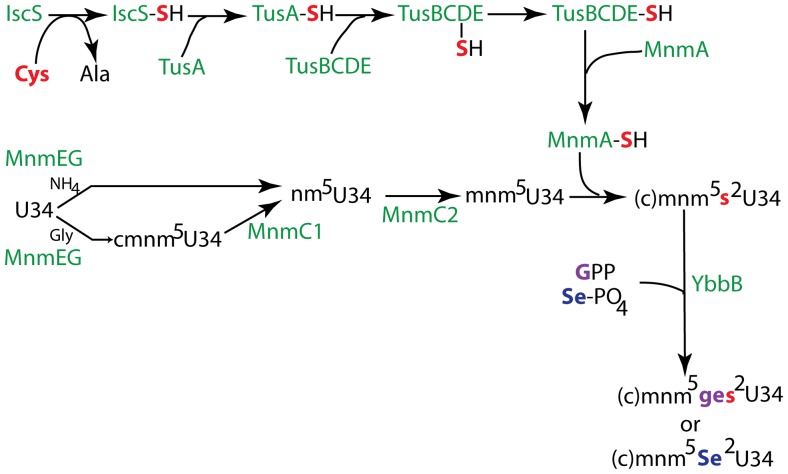
Schematic picture of the synthesis of (c)mnm^5^s^2^U34, mnm5ges^2^U34, and se^2^(c)mnm^5^U. (“ge” is a geranylgroup abbreviated “ge”; GPP is geranylpyrophosphate). The sulfur relay from Cys to the s^2^-group of the nucleoside is shown in red and the different enzymes involved in the synthesis of these thiolated derivatives are shown in green denoted as protein with their genetic symbols starting with a capital letter. A geranylgroup from GPP is transferred to cmnm^5^s^2^U of 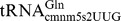 by YbbB to generate the hypermodified ges^2^cmnm^5^U34 [69] and to mnm5s2U of Lys- and Glu-tRNA to generate ges^2^ mnm^5^U [77]. YbbB is also responsible for the exchange of s^2^ by Se forming mnm^5^Se^2^U if selenium phosphate is available [89].

The first step in the synthesis of the side chain present at position 5 of wobble nucleoside (c)mnm^5^s^2^U34, which is present in tRNAs specific for Gln, Lys and Glu, is catalyzed by a heterodimer of MnmG (earlier denoted GidA) and MnmE proteins [64] (Fig. 4). This reaction generates the cmnm^5^-side chain in the presence of glycine or nm^5^-side chain in the presence of ammonia. The MnmC1 activity of MnmC (MnmC enzyme contains two activities, C1 and C2 [65]) converts the cmnm^5^-group to an nm^5^-group which in turn is converted to the mnm^5^-side chain by the MnmC2 methyltransferase activity. The cmnm^5^-side chain is present in a subset of 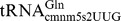 whereas 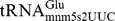 and 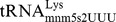 chains contain only the mnm^5^-side chain. Therefore, the synthesis of the mnm^5^-side chain depends on four enzymatic activities and any alterations of these proteins encoded by the *mnmE*, *mnmG* and *mnmC* genes might change the extent of modification of the wobble nucleoside (c)mnm^5^s^2^U34 and thereby inducing inefficient decoding.

The first step in the synthesis of the s^2^-group, also present in mnm^5^s^2^U, is catalyzed by the cysteine desulfurase IscS, whose activity is required for the synthesis of all thiolated nucleosides in bacteria [66], [67]. The IscS delivers persulfide sulfur from Cys to TusA, which in turn transfers the sulfur to tRNA in a sulfur relay system consisting of the TusBCDE complex and finally delivers the sulfur to the tRNA by MnmA [68]. Thus, the formation of the s^2^-group depends on seven proteins (IscS-TusA-TusECDE-MnmA) and alterations in any of these proteins should reduce the formation of the s^2^-group of (c)mnm^5^s^2^U34. Deficiency of the s^2^-group should negatively influence the coding capacity of the three tRNAs having (c)mnm^5^s^2^U34 as wobble nucleoside.

Of the 467 extragenic +1 frameshift suppressor mutants isolated 72 abolished, or reduced, the synthesis of (c)mnm^5^s^2^U34. This large fraction of this kind of mutants depends on the fact that 10 genes are the targets for mutations reducing the synthesis of the (c)mnm^5^s^2^U34. Several of these mutants were analyzed for the level of (c)mnm^5^s^2^U in their tRNA. Both the frameshifting phenotype and the reduced level of (c)mnm^5^s^2^U in the tRNA were returned to that of the wild type by introducing a complementing plasmid (Table 5). According to our model, reduced activity of Gln-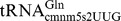 due to deficiency of the modified nucleoside should induce a shift in frame by the peptidyl-Pro-tRNA and indeed this is the case [16]. Moreover, the entry of 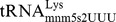, which also contains the wobble nucleoside mnm^5^s^2^U, to its cognate codon AAG in the A-site should also be reduced. We therefore determined the sequence of the frameshift peptide using plasmids pUST310 (CCC-CAA(Gln)) or pUST311(CCC-AAG(Lys)) which should monitor the slippage of peptidyl-Pro-tRNA upon slow entry of 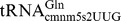 and 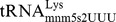, respectively (Fig. 3). The amino acid sequences of the frameshift peptide for two different *mnmA* mutants (lacking the s^2^-group) and one *mnmE* mutant (lacking the mnm^5^-sidechain) were all consistent with a frameshift error occurring at the sequence CCC-CAA or CCC-AAG by peptidyl-Pro-tRNA (Table 5). Thus, we conclude that the frameshifting phenotype of these 72 mutants lacking (c)mnm^5^s^2^U34 is consistent with our proposed model (Fig. 1, alt. C).

**Table 5:** Analysis of some typical mutations in genes inducing suppression of frameshift mutations.

Locus	Site used atselection	Alteration	mnm^5^s^2^U(% of wt)	Suppression		Complementation		P-site	Peptide (c)
				Growth withoutHis (a)	Rel b-gal(b)	FS	Rel β-gal		
**mnmA3**	D10122	G24D	<3	Yes	4.4	Yes	90	Yes^h^	p310(P)
**mnmA2**	D10111	F35S	<3	Yes	2.39	Yes	nd	Yes^h^	p311(P)
**iscS56**	C3737	S184I	38	Yes	nd	Yes	102	Yes^i^	
**tusDCB (B27)**	D10122	Q31stop	3	Yes	3.95	Yes	95	Yes^i^	
**tusCD(CD33)**	D10111	deletion	nd	Yes	nd	nd	nd		
**tusE30**	D10122	K128stop	<3	Yes	4.24	Yes	73	Yes^i^	
**tusA32**	D10122	C19Y	3	Yes	nd	Yes	104	Yes^i^	
**mnmE13**	D10122	Codon 247-271 deleted	5	Yes	1.44	Yes	88	Yes^h^	p310 (P)
**mnmG1 (gidA)**	D10122	58 nt deletion from A402	5	Yes	1.5	Yes	100	Yes^j^	
**mnmG2**	D10111	G340stop	<3	Yes	1.87	Yes	111	Yes^j^	
**hisT**	D10122	deletion	Not rel	yes	Nd.	Nd.	nd	Yes^k^	
**proL2240**	D10122	A G added in AC	Not rel	Yes	2.66	Not rel	Not rel	Yes^e^	
**proL2231**	C3737	G2A		Yes	nd	“	“	Yes^e^	
**proL2241**	D10111	G10A		Yes	nd	“	“	Yes^e^	
**proK2236**	D10110	G added in AC		Yes	nd	“	“	Yes^d^	
**proM2219**	D10111	G31A		Yes	1.78	“	“	Yes^d^	p290(P)^f^
**trmD39**	D10111	D150G	16 (m^1^G)	Yes	1.91	Yes	95 (m^1^G)	Yes^e^	p310(P); p311(F), p311 (P)
**glnU1538**	C3737	G5C	Not rel	Yes	nd	Not rel	Not rel	Yes	p290(P); p292 (F)
**glnS1544**	D10122	N70S	Not rel	Yes	nd	Yes	Not rel.	nd	nd
**rpsI2**	D10122	20 aa shorter	Not rel	Yes	2.86^f^	Yes	Not rel.	Yes^f^	p290(P)^f^; p292 (F)^f^; p310 (Pro)^f^
**rpsI3**	D10122	33 aa shorter	Not rel	Yes	3.86^f^	Yes	Not rel.	Yes^f^	p290(P)^f^; p292 (F)^f^; p310 (Pro)^f^
**ybbB181**		G67R	Reduced/newmodification	Yes	nd	No, ybbB181 is dominant		Yes^g^	p290 (P)^g^

**a)**Monitored as the ability to suppress the *his*-allele the mutant was selected to suppress. Growth of mutants on a plate lacking His following and incubation at 37°C for 4–6 days. The parental strain, which has no suppressor mutation but the indicated *his*-allele, did not grow on plates without His.

**b)**The suppression was monitored as the ability to suppress the CCC-CAA-UAG sequence placed in front of the *lacZ* gene (See M–M).

**c)**Plasmids (See figure 4) used to determine the amino acid sequence at the frameshifting site and the last amino acid in the zero frame is indicated in parenthesis.

**d**)P-site according to Qian et al 1998 [34].

**e**)P-site according to [35].

**f**)According to [36].

**g)**According to [69].

**h**)According to [16].

**i**)P-site since these mutants also lack the s^2^-group of mnm^5^s^2^U similar to the *mnmA* mutants.

**j)**P-site since *mnmG* mutants like *mnmE* mutants lack the mnm^5^-side chain of mnm5s2U.

**k)**According to [71].

pUST136: contains the *metT* operon in which *glnU* is present. It also contains the gene *miaB*. See Esberg et al 1999 [85].

pCBS4 (*glnS^+^*) contains the *glnS^+^* wild type allele.

### 4. Geranylation of (c)mnm^5^s^2^U34 Results in a Decreased Charging of (c)mnm^5^s^2^U34 Containing tRNAs and thereby Inducing a +1 Frameshift Phenotype (One Mutant)

One mutant was found to have an altered YbbB protein (G67R). The mutation is dominant and it induces the ability of the altered YbbB protein to add a geranyl-group (a C_10_H_17_-fragment abbreviated “ge”) to the sulfur of the wobble nucleoside cmnm^5^s^2^U34 of tRNAs specific for Gln [69], [70]. This generates the presence of mnm^5^ges^2^U34 in a fraction of 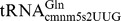 which in turn reduces the level of glutaminylated 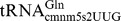. Apparently, only certain amino acid substitutions induce this activity explaining that we only found one mutant among the 467+1 frameshift mutants isolated. Interestingly, the alteration of YbbB found here (*ybbB181*,G67R) is an amino acid substitution at the same position of YbbB as a mutant (denoted *ybbB204*,G67E) isolated in 1966 and earlier characterized by us [69]. In the case of 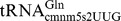 this alteration results in a decreased level of charged 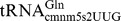 [69] nicely explaining its ability to induce +1 frameshifting. A reduced level of charged 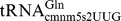 induces a pause and allows the peptidyl-Pro-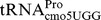 to shift frame. The resulting frameshift peptide is consistent with this interpretation [69]. Thus, the frameshifting phenotype of the YbbB (G67R) mutant was found to be consistent with the proposed model (Fig. 1, alt C).

### 5. Deficiency of Ψ38 of 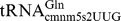 Induces +1 Frameshifts (10 Independent Mutants)

Pseudouridine (Ψ) is present in the anticodon loop and stem in positions 38, 39, and 40 in several tRNA species. The 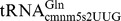 has Ψ in position 38 and 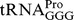, which reads the CCC codon, has Ψ40. Whereas deficiency of Ψ in position 38 reduces the rate of A-site selection of all 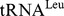:s which all have Ψ38, deficiency of Ψ40 of 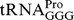 does not [71] (Reviewed in [72]). Such reduced rate of A-site selection results in a frameshift in the P-site [71]. Therefore, Ψ38 deficient 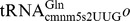 enters the A-site slowly resulting in a ribosomal pause and thereby allowing the 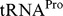 (most likely the 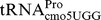) in the P-site to shift frame according to our model. We characterized 10 *hisT* mutants and found that their frameshifting phenotype is consistent with our model (Fig. 1, alt. C).

### 6. Alterations in the Activity of the Three tRNA^Pro^ :s Induce +1 Frameshifts (253 *proL*, 1 *proM* and 2 *proK* Mutants)

**Figure 5:**
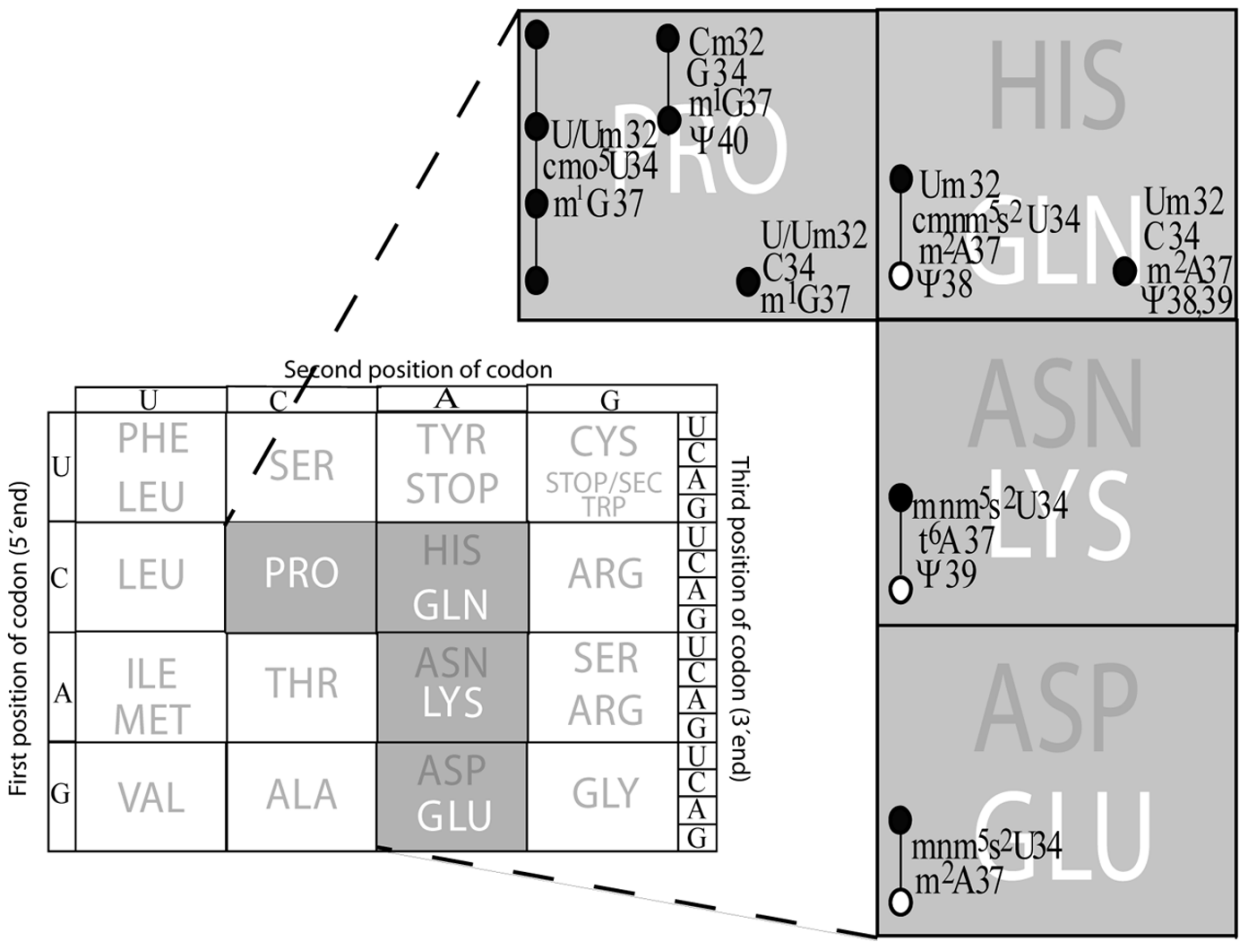
Modified nucleosides in positions 32, 34, 37, and 38–40 and the coding capacities of the corresponding tRNAs. In the proline coding box there are three tRNAs reading the four proline codons and they are encoded by *proK*, *proL*, and *proM* (One copy of each gene is present in *Salmonella*). *proM* tRNA has cmo^5^U34 as wobble base and decode all four proline codons [73]. A circle corresponds to a codon read by a tRNA and the line between circles denotes that the same tRNA read those codons. Note also that the *proM* tRNA is essential, since it is the only tRNA reading the CCA codon. The *proL* tRNA having G34 as wobble nucleoside reads U and C ending codons and *proK* tRNA, which has C34 as wobble nucleoside, should read only CCG codon. The Gln codons CAA/G are read by two tRNAs having mnm^5^s^2^U34 and C34 as their wobble nucleoside. The C34 containing tRNA reads only CAG whereas the mnm^5^s^2^U containing tRNA (*glnU* tRNA) decodes both CAA and CAG although less efficient CAG (Unfilled circle). Note that the latter tRNA *(glnU* tRNA) is essential, since it is the only tRNA reading the CAA codon. In the Lys and Glu codon boxes one tRNA having mnm^5^s^2^U as wobble nucleoside reads AAA (Lys)/GAA (Gln) and less efficient AAG (Lys)/GAG (Glu) (Unfilled circle).

The four proline codons are read by *proL* (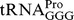), *proM* (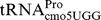) and *proK* (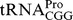) and their coding capacities are shown in Figure 5. The *proM* 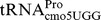 reads all four proline codons and is the only tRNA^Pro^ that read CCA [73]. Accordingly, the *proM* tRNA is essential for viability and no frameshift suppressor mutant has earlier been isolated as having an altered *proM* tRNA. The *proL* 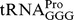 has anticodon GGG and reads the codons CCC and CCU and the *proK* 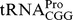 with its anticodon CGG reads only CCG. The classical dominant +1 frameshift suppressors are derivatives of *proL* (*sufB2*) and of *proK* (*sufA6*). Both these frameshift suppressors have an extra base inserted in the anticodon loop [34], [74]. We isolated 253+1 frameshift suppressor mutants, which were in some way defective in the synthesis or activity of *proL* 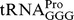 (Table 5). This large amount of *proL* mutants was expected, since the *proL* gene, is not essential for viability and deletion of this gene induces +1 frameshifting [75]. Table 6 shows that deletions, duplications, base substitutions and promoter mutations were obtained. Only three mutants (two in *proK* and one in *proM*) were obtained that affected the other two tRNA^Pro^:s. Since the *proM* is essential we did not expect many mutants defective in this gene especially as no +1 frameshift mutant has earlier been characterized (See Discussion). However, the *proK* is not essential [73] and we expected an equally large amount of mutations in this gene as in *proL*. However, this was not the case and moreover, the two independently isolated *proK* mutants have an insertion of a G in the anticodon similar to the classical *sufA6* tRNA^Pro^ [34]. A deletion of the *proK* gene does not induce +1 frameshifts of either the *hisD10122* nor the *hisC3737* mutations ([76] and unpublished results). The small number of *proK* mutants suggests that only specific alterations of the *proK* gene induce frameshifting and this aspect is discussed below (See Discussion).

**Table 6:** +1 frameshift suppressor mutants lacking or with defects in the *proL* 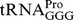.

His-alleles used for selection		*C3737*	*C10106*	*D10110*	*C10109*	*D10122*	*D10111*	
Mutations	Alterations in tRNA regions	ccc- caa	ccc- aug	ccc-uau	ccc-ugg	ccc- caa	ccc-aag	
Not sequenced^a)^	**N/D**	**3**				85	38	126
*proL::*Tn*10*	N/R	11						11
*proL*::EZ	N/R		44		5			49
G2A	AA-stem	1						1
G2A, G-2A	”	1						1
G7A	”	1						1
G72A	”	1						1
G53A	TΨ-loop	1						1
G10A	D-stem	1			8			9
C11U	D-stem		1					1
G15A	D-loop				1			1
G19U	D-loop			2				2
G20A	D-loop				1			1
dupl G42-C48	AC-stem		2					2
dupl G36-C42 (U39-G44)	AC-loop and and stem		1					1
del G40-G46(G45-G51)	AC-stem		1					1
del U40	AC-stem			1				1
G67U	AA-stem			1				1
delG36-C42	AC-stem			4				4
delG39-G45	AC-stem				4			4
G29A	AC-stem				1			1
dupl G44-G50	AC-stem				1			1
G36A	AC				1			1
extra G in the anticodon	AC	1		1				2
del G in anticodon	AC		1					1
del G35-C41	AC-loop		7		20			27
promoter mutation −35					1			1
								0
**Total**		**21**	**57**	**9**	**43**	**85**	**38**	**253**

a)These mutations were not sequenced but located by transduction with a transposon closely linked to the indicated gene. The linkage between the transposon and the mutation was consistent with it being in the *proL* gene.

The 256 characterized mutations altering a proline tRNA induce +1 frameshift errors. For *proL* mutants it has been shown earlier that the frameshift occurs in the P-site [35] and this is the case also for mutants in *proK* [34] and in *proM* [36]. Thus, the frameshifting phenotypes of all these 256 mutants defective in any of the three tRNA^Pro^ are all consistent with the frameshifting model [Fig. 1, Alt. A (*proL* and *proK*) and B (*proK* and *proM*)].

### 7. m^1^G37 Deficiency Induces +1 Frameshift in the P-site (19 Mutants)

The *trmD* gene codes for the enzyme (tRNA(m^1^G37)methyltransferase, which synthesizes m^1^G37 in all proline tRNAs, all leucine tRNAs reading the CNN codons, and the arginine tRNA reading codon CGG. We obtained 19 *trmD* mutants of which 6 were sequenced and tested for the level of m^1^G in their tRNAs. As expected, very low levels of m^1^G were present in their tRNA (Table 6). Lack of m^1^G37 was the first modification deficiency shown to induce frameshift errors [18] and such deficiency induces frameshift errors in the P-site [35] consistent with our model (Fig. 1, alt. B).

### 8. Physical Alterations in the Ribosomal P-site Induce +1 Frameshifts (Two Mutants)

**Figure 6:**
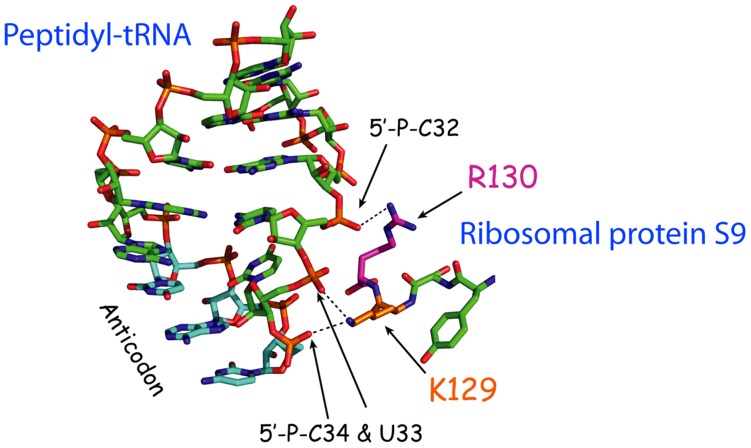
The anticodon loop of the peptidyl-tRNA and the extreme C-terminal end of ribosomal protein S9. The anticodon of the peptidyl-tRNA is labeled blue, the C-terminal Arg130 of S9 is in purple and Lys129 is in orange. The arrow points to the 5′ phosphate of the wobble nucleotide pC34. The dashed line indicates a possible H-bond between the phosphate of pC34 and the amino group of Lys129 [6]. The figure is adopted from [36] with permission.

Alterations of the ribosomal P-site might also induce +1 frameshifts if the ribosomal grip of the peptidyl-tRNA is weakened. The C-terminal end of ribosomal protein S9 penetrates the ribosome like a tentacle and the two last amino acids make a contact with the 5′ phosphate of nucleotide 32 (R130) and the 5′-phosphates of positions 33 and 34 (K129) of peptidyl-tRNA (Fig. 6; [6]). Thus, ribosomal protein S9 might be an important feature of the ribosomal grip of the peptidyl-tRNA in order to maintain the reading frame. Indeed, two of the 467 independently isolated +1 frameshift mutants (*rpsI2* and *3*) had a 20 amino acids or a 33 amino acids, respectively, truncated C-terminal of ribosomal protein S9. Analysis of these mutants as well as two mutants isolated by direct substitution of amino acid R130 and K129 revealed that the frameshift occurred in the P-site as shown both by peptide sequencing of the frameshift product and by reduced frameshift by overexpression of the aa-tRNA predicted to read the A-site codon [36]. The unexpected isolation of these two +1 frameshift mutants having a defective ribosomal protein S9 among all our 467 independently isolated +1 frameshift suppressor mutants strongly support the fundamental importance of the ribosomal P-site in maintaining the reading frame.

## Discussion

Maintaining the reading frame is a vital feature of translation in all organisms. Here we extensively test a model for how +1 frameshift errors may occur (Fig. 1). A key feature of the model is the critical role of interactions between constituents of the ribosomal P-site and the peptidyl-tRNA in maintaining the reading frame. If a ternary complex consisting of aa-tRNA, EF-Tu and GTP, is slow reading the codon in the A-site, the probability of destructions of interactions between the peptidyl-tRNA and some key components of the ribosomal P-site increases and a frameshift error may occur by the peptidyl-tRNA. We have characterized many (467) independently isolated mutants able to suppress a +1 frameshift mutation; i.e mutants with defects in maintaining the reading frame. Analyses of the frameshifting phenotype of all these mutants can be explained by our model. The mutants were defective in 16 different loci of which 15 in various ways reduced the activity of a tRNA and thereby changed either the rate with which the ternary complex enters the A-site or its interaction as part of the peptidyl-tRNA with some P-site component(s). The model also predicts that some alterations of the P-site environment might mediate a slippage of wild type peptidyl-tRNA. Indeed, we obtained two such +1 frameshifting mutants which possessed an altered ribosomal protein S9, whose C-terminal reaches into the P-site and interacts with the anticodon of the peptidyl-tRNA (Fig. 6). The fact that we obtained these S9 mutants as able to suppress +1 frameshift mutations is a strong support for the pivotal role of the P-site in maintaining the reading frame.

At certain frameshifting sites (Table 2) the glutamine codon CAA was present. This codon is read by 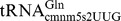 encoded by the *glnU* gene, which is found in only one copy in *Salmonella.* It is thus essential for viability. Using such sites to select for frameshift suppressor mutants, we expected to find only mutants with a reduced level, charging, or stability of this tRNA, but no deletion of its only structural gene *glnU*. This was indeed the case and we obtained 106 mutants of which we sequenced 23 and analyzed 12 in detail (Fig. 2; Table 3 and 4). Using the two frameshift mutations *hisC3737* and *hisD10122*, both having the frameshift site CCC-CAA, these kinds of mutants were frequent, suggesting that deficiency of a fully active 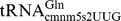 results in efficient frameshifting, explaining the ease with which we obtained them. The 12 mutants analyzed in detail were either temperature or cold sensitive. All of them charged the 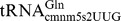 efficiently, but the level of Gln-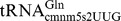 was decreased due to instability of the tRNA (Fig. 2). According to our model a reduced availability of Gln-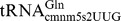 should induce a shift in frame by tRNA^Pro^ in the P-site. Indeed this was shown by sequencing the frameshift product and the expected amino acid was consistent with the tRNA^Pro^ in the P-site having shifted frame (Fig. 3, plasmid pUST290). Moreover, to show that a lower level of 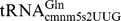 could also induce another peptidyl-tRNA to shift frame, we determined the frameshift product of a UUU-CAA site (Fig. 3, plasmid pUST292). Such an analysis showed that the tRNA^Phe^ shifted frame. Thus, we conclude that the frameshift phenotype of all these *glnU* mutants is consistent with our model (Fig. 1). The 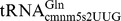 is charged by Gln-tRNA synthetase (GlnRS), which is encoded by the *glnS* gene. Defective GlnRS results in reduced charging of 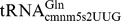 but we expected to find only a few mutations in this gene since it is essential explaining why we find only one such mutant.

The 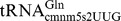 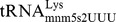 and 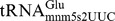 have as wobble nucleoside (c)mnm^5^s^2^U34, the complex synthesis of which is shown in Figure 4. Neither the s^2^- nor the (c)mnm^5^-group are essential for growth although deficiency of any of them severely reduces the growth. We have earlier shown that lack of this wobble modification induces +1 frameshifting [16]. Since synthesis of this modification requires as many as 10 genes, we expected many mutants defective in the synthesis of (c)mnm^5^s^2^U as was the case. The 72 independently isolated mutants were obtained using either the *hisD10122*/*C3737* (CCC-CAA) or the *hisD10111* (CCC-AAG) frameshift mutations. Deficiency of the s^2^-group or the mnm^5^-group induces frameshifting irrespectively of which gene involved in their synthesis is affected. This shows that it is the undermodified tRNA that is responsible for the frameshift phenotype and not lack of any of the biosynthetic proteins involved in the synthesis of these modifications. Determination of the frameshift products encoded both at a CCC-CAA and CCC-AAG site, showed that it was the P-site tRNA^Pro^ that slipped into the +1 frame (Fig. 3; pUST310 and pUST311; Table 6). Thus, these 72 independently isolated mutants defective in the synthesis of mnm^5^s^2^U induce a +1 frameshift by peptidyl-tRNA slippage caused by slow entry of the mnm^5^s^2^U34 deficient tRNA specific for Gln or Lys consistent with the model presented in Figure 1 (Alt. C).

We also obtained one mutant with an amino acid alteration at position 67 of the YbbB protein and such alteration increases the activity of the YbbB protein to transfer a geranyl (“ge”) group to the s^2^-group of cmnm^5^s^2^U in 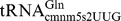 generating a ges^2^cmnm^5^U hypermodified 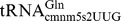 [69], [70]. This hypermodification decreases the glutaminylation of the geranylated 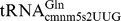 [69], [77]. As the sequence of the *trans*-frame encoded peptide revealed, it induces +1 frameshifting in the P-site [69]. Such alteration of the YbbB protein also mediates geranylation of the Glu- and Lys-tRNA, which contain the mnm^5^s^2^U as wobble nucleoside [77]. Unlike the hypermodified Gln-tRNA, the hypermodified tRNA^Lys^ and tRNA^Glu^ are aminoacylated to the same level as the respective wild type tRNA and still a +1 frameshift occurs at a +1 frameshift mutation containing lysine codons [77]. Since the frameshift peptide was not established it is not known which tRNA makes the frameshift error. However, a ternary complex having a tRNA with such a large hydrophobic compound as part of the wobble nucleoside is not likely to be efficiently accepted, if at all, in the A-site. This causes a ribosomal pause and induces the peptidyl-tRNA to make a +1 frameshift error. Thus, the +1 frameshifting induced by geranylated Lys-tRNA may be consistent with our model (Fig. 1 alt C).

The *proL* gene is not essential for viability and deletion of this gene also mediates +1 frameshift suppression [36], [75]. Therefore, we expected to obtain many mutations in the *proL* gene, as we indeed did (Table 3). Since we obtained deletions, duplications, promoter mutations as well as various base substitutions in this gene, it appears that any alteration that reduces the activity of 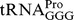 mediated +1 frameshifting (Table 5) consistent with the observation that a deletion of the *proL* gene induces +1 frameshifting. Note, that one of the *proL* mutations had an extra G in the anticodon and thus was identical to the classical *sufB2* mutation obtained in 1970 [46], [74]. Genetically it is possible to remove the cmo^5^U modification, which is only present in the *proM* 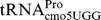 among the tRNA^Pro^:s. Such manipulation reduces, by almost 100%, the *proL* mediated +1 frameshift suppression, demonstrating that it is the wild type *proM* 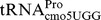 that makes the frameshift error and not the altered *proL* 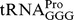 [34]. Although 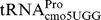 is able to read the CCC codon [73], its interaction with the near-cognate codon CCC in the P-site may not be optimal thus inducing a +1 slippage upon a ribosomal pause. Since the frameshifting event occurs in the P-site [35], the many mutants obtained with defects in the *proL* gene is consistent with our model (Fig. 1, alt A).

The *proM* 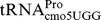 is essential for growth and no alterations of this tRNA have been described earlier. We therefore did not expect many mutants with altered *proM* 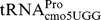. The one we obtained (G31A) disrupted the last base-pair of the anticodon stem creating a 9 member anticodon loop. Using localized mutagenesis we have isolated additional 108 mutants all with point mutations in the *proM* gene [36]. Among these 109 mutants, none had an extra base in the anticodon as the classical +1 frameshift derivatives of *proL* (*sufB2*) and *proK* (*sufA6*) have. The G31A mutation was found in 32% of these *proM* mutants most likely because it is the strongest frameshift suppressor among the 108 *proM* mutants characterized. Interestingly, most of the alterations in the *proM* 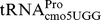 are in close proximity to components in the ribosomal P-site further supporting the pivotal role of the P-site in reading frame maintenance [36]. Accordingly, the frameshift error induced by these altered *proM* 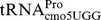: s occurs in the P-site as determined by amino acid sequence of the frameshift product and from overexpression of the tRNA reading the A-site codon [36]. Clearly, our extensive selection of +1 frameshift suppressor mutants identified novel alterations in the tRNA^Pro^ family and identified, for the first time, an alteration of the *proM* 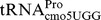 that mediates a frameshift error with a mechanism consistent with our model (Fig. 1, alt. B).

In *Salmonella* 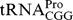 is encoded by a single gene, *proK*, and with its C34 as wobble nucleoside it should only read CCG (Fig. 5). Although a mutant with a *proK* deletion is viable and grows as wild type such a strain does not suppress the *hisC3737* or the *hisD10122* mutations (unpublished observation). Apparently only specific changes of the *proK* 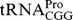 induce +1 frameshift suppression consistent with a *proK* mutation (*sufA6*) being dominant [78]. We therefore did not expect many +1 frameshift suppressor mutants with alterations in this tRNA. Indeed, this was the case, since only two independently isolated mutants (*proK2236* and *proK2237*) with an altered *proK* 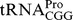
were obtained. Interestingly, both of them had the same alteration as the classical *sufA6* mutant; i.e an extra G in the anticodon loop. The *sufA6* tRNA, and therefore the two altered *proK* 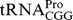:s characterized here, have a normal sized anticodon bordered by m^1^G37 and U33 and the insertion of the extra G is 3′ to the m^1^G37 [34]. Such altered *proK* tRNA induces frameshifting in the P-site according to our model [34]. The three-nucleotide size of the anticodon of these *proK* mutants and their induction of +1 slippage in the P-site are not consistent with the quadruplet translocation model. Such *proK* mutants have three tRNA^Pro^:s (wild type *proL* 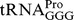
and *proM* 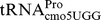 in conjunction with the altered *proK* 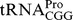) any of which may cause the +1 frameshift error and thereby suppress the +1 frameshift mutation. Of these, the *proL* 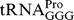 is the cognate tRNA reading the CCC codon and we therefore find it unlikely that this tRNA makes the frameshift error in the P-site, since its structural interaction with the P-site environment and potential anticodon-codon interaction, is normal. Thus, either the wild type *proM* 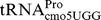 or the altered *proK* 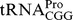 causes the frameshift error. Removing the cmo^5^U modification genetically reduces the frameshift suppression by about 50% suggesting that this part of the frameshifting is due to wild type *proM* 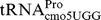, since it is the only tRNA^Pro^ containing this modified nucleoside [34] (Fig. 1, alt A). As the s*ufA6* (a derivative of *proK*) mediated frameshifting occurs in the P-site [34], it is apparently a mixed population of peptidyl-Pro-tRNA^Pro^ (wild type *proM* and mutant *proK* (*sufA6, proK2236,* or *proK2237*) tRNAs) that slips into the +1 frame in the P-site. The *proM* 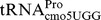 is less abundant in the cell compared to *proL* and *proK* tRNAs (68% of *proL* and 95% of *proK* at growth rate of 2.5 doublings [79]). In the *proK* mediated frameshifting, *proM* 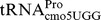 must therefore compete efficiently with the wild type *proL* 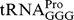 in reading the CCC codon, since 50% frameshifting is caused by the *proM* 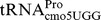 [34]. The residual 50% of frameshifting occurring in the P-site must therefore be caused by the altered *proK* 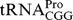 (Fig. 1, alt B). The distance between the two strands of the tRNA and mRNA in the anticodon-codon interaction is too large for two pyrimidines (C34 of *proK* 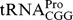 and C(III) in the codon) to interact. Consequently it is not likely that *proK* 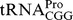 makes a pairing with the third nucleoside (CIII) of the CCC codon. We therefore propose that the altered *proK* 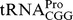 with its extra G in the anticodon loop, but still having a normal sized anticodon bordered between m^1^G37 and U33 [34], is able to make 2 out of 3 interaction with the CCC codon in the A-site. After a three nucleotide interaction, its fitting in the P-site is not optimal making it prone to shift frame. Since the *proL* 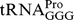 is present in the cell at about the same concentration as the altered *proK* 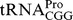 such a 2 out of 3 interaction can compete with a cognate interaction by the wild type *proL* 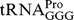 to read the CCC codon consistent with the altered *proK* 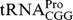 being dominant. The fact that we found two *proK* mutants with the same alteration as the classical *sufA6*+1 frameshift mutant is consistent with our model since the frameshift event occurs in the P-site [34].

As pointed out above, deletion of *proK* is viable but does not suppress the *hisC3737* (CCC-CAA) or *hisD10122* (CCC-CAA). However, in the presence of the *proK* mutant tRNA with an extra G in the anticodon loop, 50% of the suppressor activity is due to the wild type *proM* 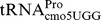 (Fig. 1, alt A). From this result one would expect that a deletion of *proK* should allow *proM* 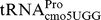 mediated frameshifting. Why is this not occurring? Apparently the *proM* 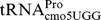 is able to read the CCC codon in the presence of mutant *proK* tRNA but not when the wild type *proK* 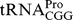 is absent. The reason may be that in a cell having *proM^+^* and *proL^+^* tRNAs, like in a mutant with *proK* deleted, the only tRNA reading the CCG codon is *proM* 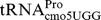. This may result in too low a concentration of the *proM* 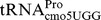 to compete with the *proL* 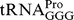 in reading the CCC codon explaining that a deletion of the *proK* gene does not induce a frameshift by the *proM* 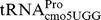.

From above we conclude that the ability to decode the four proline codons depends not only on the decoding capacities as revealed by the anticodon-codon interaction but also on the availability of the different Pro-tRNAs to decode the various proline codons in the cell at various conditions. Clearly, the analysis of the +1 frameshift phenotypes of the *proK*, *proL*, and *proM* mutants have revealed an intricate competition between the three Pro-tRNA^Pro^:s to read the CCC codon and thereby induce +1 frameshifting in the P-site at the various frameshift mutations used.

All three tRNA^Pro^ have m^1^G37 next to and 3′ of the anticodon. Lack of m^1^G37 results in a tRNA^Pro^ having three consecutive G:s in the anticodon loop. When it was discovered that lack of m^1^G induces +1 frameshifts [18], it was suggested that such m^1^G deficient tRNA^Pro^ therefore had the possibility of making a four base interaction in the A-site and by a quadruplet translocation move the tRNA to the P-site. However, the m^1^G deficient tRNA slips forward one nucleotide in the P-site [35] which is not consistent with the quadruplet translocation model but is consistent with the model presented here (Fig. 1). The rate with which the three tRNA^Pro^:s enter the A-site is reduced, by 50–90%, due to the lack of m^1^G [71]. Of the three tRNA^Pro^:s, the *proL* 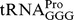 is the least affected (50%) by m^1^G deficiency. It is the cognate tRNA for the CCC codon and its concentration in the cell is larger than that of *proM* 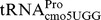, which also reads CCC. Therefore we suggest that it is mainly the m^1^G deficient cognate *proL* 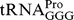 which makes a slippage in the P-site due to the lack of m^1^G although we cannot exclude that also the m^1^G deficient *proM* 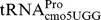 makes such an error. The m^1^G37 may interact in the P-site with some constituents of rRNA/r-protein(s), and lack of it in *proL* 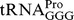 or in *proM* 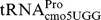, may disrupt such a stabilizing interaction of the peptidyl-tRNA. Lack of the ms^2^-group or of the ms^2^io^6^-groups of the ms^2^io^6^A37, which is, like m^1^G37, present in position 37 but in tRNA^Phe^ and tRNA^Tyr^, also induces +1 slippage in the P-site [16]. Crystal studies of tRNA^Phe^ show that the ms^2^i^6^A37 is part of a network of interactions with the first base of the P-site codon, third base of the E-site codon and anchors the tRNA in the P-site with A790 and U789 of the 16S rRNA [80]. Thus, these structural data support a pivotal role of the P-site environment to maintain the reading frame and more specifically point to some fundamental interactions between position 37 of the peptidyl-tRNA and 16S rRNA. Interestingly, lack of two other modified nucleosides (t^6^A37 and yW37) present in position 37 of the tRNA also mediate increased frameshifting [21]–[23]. Apparently, the modification in position 37 of several tRNAs is important to maintain the reading frame. In summary, the fact that m^1^G deficiency induces frameshift errors in the P-site further supports the model in Figure 1 and the structural data presented for another modified nucleoside present in the same position as m^1^G, strengthen the importance of the P-site environment in reading frames maintenance and the role of modification in position 37 of tRNA in this process.

If the ribosomal P-site is important for reading frame maintenance, one would expect that a nonessential alteration in this part of the ribosome would also induce +1 frameshifting. Of course we did not expect many mutants defective in this part of the ribosome since many of such alterations would be lethal due to the important function of the P-site in translation. Still, we obtained two mutants among the 467+1 frameshift mutants both of which lacked several amino acids of the C-terminal end of ribosomal protein S9. The S9 protein is part of the common core shared by bacteria and eukaryotes and is one of the 34 conserved ribosomal proteins (15 in the small subunit and 19 in the large subunit) [81]. The C-terminal part of the S9 protein penetrates like a tentacle into the P-site of the ribosome and the two last amino acids of the protein make contacts with the 5′-phosphate of nucleotide 32 (R130) and the 5′-phosphates of positions 33 and 34 (K129) of peptidyl-tRNA [6] (Fig. 6). The existence of these mutants prompted us to change the two most C-terminal amino acids of S9 protein and monitor the ability to induce +1 frameshifts [36]. Indeed all these *rpsI* (S9) mutants induce +1 frameshifts. The isolation and characterization of these mutants defective in ribosomal protein S9 is a strong support that the frameshift event occurs in the P-site and makes the ribosomal grip of the peptidyl-tRNA one of the important features to maintain the reading frame.

### Conclusion

All our results support the frameshifting model presented in Figure 1 and demonstrate the pivotal role of the ribosomal grip of the peptidyl-tRNA. Moreover, the results demonstrate an intricate competition between the three Pro-tRNAs to read the four codons in the Pro-box and highlight the importance of the modified nucleosides in positions 37 (next to and 3′ of the anticodon), 34, and 38 in maintaining the reading frame.

## References

[1] Woese CR (2002) On the evolution of cells. Proc Natl Acad Sci U S A 99: 8742–8747. 12077305 10.1073/pnas.132266999 PMC124369

[2] Parker J (1989) Errors and alternatives in reading the universal genetic code. Microbiological Reviews 53: 273–298. 2677635 10.1128/mr.53.3.273-298.1989 PMC372737

[3] Kramer EB, Farabaugh PJ (2007) The frequency of translational misreading errors in E. coli is largely determined by tRNA competition. RNA 13: 87–96. 17095544 10.1261/rna.294907 PMC1705757

[4] Kramer EB, Vallabhaneni H, Mayer LM, Farabaugh PJ (2010) A comprehensive analysis of translational missense errors in the yeast Saccharomyces cerevisiae. RNA 16: 1797–1808. 20651030 10.1261/rna.2201210 PMC2924539

[5] Kurland CG (1992) Translational accuracy and the fitness of bacteria. Annu Rev Genet 26: 29–50. 1482115 10.1146/annurev.ge.26.120192.000333

[6] Selmer M, Dunham CM, Murphy FV, Weixlbaumer A, Petry S, et al (2006) Structure of the 70S ribosome complexed with mRNA and tRNA. Science 313: 1935–1942. 16959973 10.1126/science.1131127

[7] Korostelev A, Trakhanov S, Laurberg M, Noller HF (2006) Crystal Structure of a 70S Ribosome-tRNA Complex Reveals Functional Interactions and Rearrangements. Cell 126: 1065–1077. 16962654 10.1016/j.cell.2006.08.032

[8] Ogle JM, Ramakrishnan V (2005) Structural insights into translational fidelity. Annu Rev Biochem 74: 129–177. 15952884 10.1146/annurev.biochem.74.061903.155440

[9] Atkins JF, Björk GR (2009) A gripping tale of ribosomal frameshifting: extragenic suppressors of frameshift mutations spotlight P-site realignment. Microbiol Mol Biol Rev 73: 178–210. 19258537 10.1128/MMBR.00010-08 PMC2650885

[10] Farabaugh PJ (1996) Programmed translational frameshifting [review]. Microbiological Reviews 60: 103–134. 8852897 10.1128/mr.60.1.103-134.1996 PMC239420

[11] Farabaugh PJ (1997) Programmed Alternative Reading of the Genetic code. Austin: R. G. Landes Company.

[12] Atkins JF, Baranov PV, Fayet O, Herr AJ, Howard MT, et al (2001) Overriding standard decoding: Implications of recoding for ribosome function and enrichment of gene expression. Cold Spring Harb Symp Quant Biol 66: 217–232. 12762024 10.1101/sqb.2001.66.217

[13] Atkins JF, Weiss RB, Thompson S, Gesteland RF (1991) Towards a genetic dissection of the basis of triplet decoding, and its natural subversion: programmed reading frame shifts and hops. Annu Rev Genet 25: 201–228. 1812806 10.1146/annurev.ge.25.120191.001221

[14] Dale T, Uhlenbeck OC (2005) Amino acid specificity in translation. Trends Biochem Sci 30: 659–665. 16260144 10.1016/j.tibs.2005.10.006

[15] Fahlman RP, Dale T, Uhlenbeck OC (2004) Uniform binding of aminoacylated transfer RNAs to the ribosomal A and P sites. Mol Cell 16: 799–805. 15574334 10.1016/j.molcel.2004.10.030

[16] Urbonavicius J, Qian Q, Durand JM, Hagervall TG, Björk GR (2001) Improvement of reading frame maintenance is a common function for several tRNA modifications. EMBO J 20: 4863–4873. 11532950 10.1093/emboj/20.17.4863 PMC125605

[17] Hagervall TG, Tuohy TM, Atkins JF, Björk GR (1993) Deficiency of 1-methylguanosine in tRNA from *Salmonella typhimurium* induces frameshifting by quadruplet translocation. J Mol Biol 232: 756–765. 7689113 10.1006/jmbi.1993.1429

[18] Björk GR, Wikström PM, Byström AS (1989) Prevention of translational frameshifting by the modified nucleoside 1-methylguanosine. Science 244: 986–989. 2471265 10.1126/science.2471265

[19] Lecointe F, Namy O, Hatin I, Simos G, Rousset JP, et al (2002) Lack of pseudouridine 38/39 in the anticodon arm of yeast cytoplasmic tRNA decreases in vivo recoding efficiency. J Biol Chem 277: 30445–30453. 12058040 10.1074/jbc.M203456200

[20] Carlson BA, Kwon SY, Chamorro M, Oroszlan S, Hatfield DL, et al (1999) Transfer RNA modification status influences retroviral ribosomal frameshifting. Virology 255: 2–8. 10049815 10.1006/viro.1998.9569

[21] Carlson BA, Mushinski JF, Henderson DW, Kwon SY, Crain PF, et al (2001) 1-methylguanosine in place of Y base at position 37 in phenylalanine tRNA is responsible for its shiftiness in retroviral ribosomal frameshifting. Virology 279: 130–135. 11145896 10.1006/viro.2000.0692

[22] Waas WF, Druzina Z, Hanan M, Schimmel P (2007) Role of a tRNA base modification and its precursors in frameshifting in eukaryotes. J Biol Chem 282: 26026–26034. 17623669 10.1074/jbc.M703391200

[23] El Yacoubi B, Hatin I, Deutsch C, Kahveci T, Rousset JP, et al (2011) A role for the universal Kae1/Qri7/YgjD (COG0533) family in tRNA modification. EMBO J 30: 882–893. 21285948 10.1038/emboj.2010.363 PMC3049207

[24] Gallant J, Lindsley D, Masucci J (2000) The unbearable lightness of peptidyl-tRNA. In: Garrett RA, Douthwaite SR, Liljas A, Matheson AT, Moore PB et al.., editors. The Ribosome: Structure, Function, Antibiotics, and Cellular Interaction. Washington, D.C.: American Society for Microbiology. 385–396.

[25] ÓConnor M (1998) TRNA imbalance promotes −1 frameshifting via near-cognate decoding. J Mol Biol 279: 727–736. 9642056 10.1006/jmbi.1998.1832

[26] Atkins JF, Gesteland RF, Reid BR, Anderson CW (1979) Normal tRNAs promote ribosomal frameshifting. Cell 18: 1119–1131. 391405 10.1016/0092-8674(79)90225-3

[27] Spanjaard RA, van Duin J (1988) Translation of the sequence AGG-AGG yields 50% ribosomal frameshift. Proc Natl Acad Sci U S A 85: 7967–7971. 3186700 10.1073/pnas.85.21.7967 PMC282334

[28] Weiss RB, Dunn DM, Atkins JF, Gesteland RF (1987) Slippery runs, shifty stops, backward steps, and forward hops: −2, −1, +1, +2, +5, and +6 ribosomal frameshifting. Cold Spring Harb Symp Quant Biol 52: 687–693. 3135981 10.1101/sqb.1987.052.01.078

[29] Weiss RB, Dunn DM, Atkins JF, Gesteland RF (1990) Ribosomal frameshifting from −2 to +50 nucleotides. Prog Nucleic Acid Res Mol Biol 39: 159–183. 2247607 10.1016/s0079-6603(08)60626-1

[30] Stahl G, Salem SN, Chen L, Zhao B, Farabaugh PJ (2004) Translational accuracy during exponential, postdiauxic, and stationary growth phases in Saccharomyces cerevisiae. Eukaryot Cell 3: 331–338. 15075263 10.1128/EC.3.2.331-338.2004 PMC387642

[31] Wenthzel AM, Stancek M, Isaksson LA (1998) Growth phase dependent stop codon readthrough and shift of translation reading frame in Escherichia coli. FEBS Lett 421: 237–242. 9468314 10.1016/s0014-5793(97)01570-6

[32] Roth JR (1981) Frameshift Suppression. Cell 24: 601–602. 6166384 10.1016/0092-8674(81)90086-6

[33] Spirin AS (1986) Ribosome structure and protein biosynthesis. Menlo Park: The Benjamin/Cummings Publishing Company, Inc.

[34] Qian Q, Li JN, Zhao H, Hagervall TG, Farabaugh PJ, et al (1998) A new model for phenotypic suppression of frameshift mutations by mutant tRNAs. Mol Cell 1: 471–482. 9660932 10.1016/s1097-2765(00)80048-9

[35] Qian Q, Björk GR (1997) Structural alterations far from the anticodon of the tRNA^Pro^ _GGG_ of *Salmonella typhimurium* induce +1 frameshifting at the peptidyl-site. J Mol Biol 273: 978–992. 9367785 10.1006/jmbi.1997.1363

[36] Näsvall SJ, Nilsson K, Björk GR (2009) The ribosomal grip of the peptidyl-tRNA is critical for reading frame maintenance. J Mol Biol 385: 350–367. 19013179 10.1016/j.jmb.2008.10.069

[37] Baranov PV, Gesteland RF, Atkins JF (2004) P-site tRNA is a crucial initiator of ribosomal frameshifting. RNA 10: 221–230. 14730021 10.1261/rna.5122604 PMC1370534

[38] Farabaugh PJ, Björk GR (1999) How translational accuracy influences reading frame maintenance. EMBO J 18: 1427–1434. 10075915 10.1093/emboj/18.6.1427 PMC1171232

[39] Sundararajan A, Michaud WA, Qian Q, Stahl G, Farabaugh PJ (1999) Near-cognate peptidyl-tRNAs promote+1 programmed translational frameshifting in yeast. Mol Cell 4: 1005–1015. 10635325 10.1016/s1097-2765(00)80229-4

[40] Harger JW, Meskauskas A, Dinman JD (2002) An “integrated model” of programmed ribosomal frameshifting. Trends Biochem Sci 27: 448–454. 12217519 10.1016/s0968-0004(02)02149-7

[41] Stahl G, McCarty GP, Farabaugh PJ (2002) Ribosome structure: revisiting the connection between translational accuracy and unconventional decoding. Trends Biochem Sci 27: 178–183. 11943544 10.1016/S0968-0004(02)02064-9 PMC7126812

[42] Bertani G (1951) Studies on Lysogenesis. J Bacteriol 62: 293–300. 14888646 10.1128/jb.62.3.293-300.1951 PMC386127

[43] Vogel HJ, Bonner DM (1956) Acetylornithinase of *Escherichia coli*: Partial purification and some properties. J Biol Chem 218: 97–106. 13278318

[44] Davis W, Botstein D, Roth JR (1980) A manual for genetic engineering: Advanced Bacterial Genetics. New York: Cold Spring Harbor Laboratory.

[45] Schmieger H (1972) Phage *P22*-mutants with increased or decreased transduction abilities. Molecular & General Genetics 119: 75–88. 4564719 10.1007/BF00270447

[46] Riddle DL, Roth JR (1970) Suppressors of frameshift mutations in *Salmonella typhimurium* . J Mol Biol 54: 131–144. 4321728 10.1016/0022-2836(70)90451-1

[47] Yourno J, Tanemura S (1970) Restoration of in-phase translation by an unlinked suppressor of a frameshift mutation in *Salmonella typhimurium* . Nature 225: 422–426. 4903822 10.1038/225422a0

[48] Maloy SR, Nunn WD (1981) Selection for loss of tetracycline resistance by *Escherichia coli* . J Bacteriol 145: 1110–1111. 7007341 10.1128/jb.145.2.1110-1111.1981 PMC217228

[49] Milton DL, O’Toole R, Horstedt P, Wolf-Watz H (1996) Flagellin A is essential for the virulence of Vibrio anguillarum. J Bacteriol 178: 1310–1319. 8631707 10.1128/jb.178.5.1310-1319.1996 PMC177804

[50] Wagner J, Nohmi T (2000) Escherichia coli DNA polymerase IV mutator activity: genetic requirements and mutational specificity. J Bacteriol 182: 4587–4595. 10913093 10.1128/jb.182.16.4587-4595.2000 PMC94631

[51] Wagner J, Gruz P, Kim SR, Yamada M, Matsui K, et al (1999) The dinB gene encodes a novel E. coli DNA polymerase, DNA pol IV, involved in mutagenesis. Mol Cell 4: 281–286. 10488344 10.1016/s1097-2765(00)80376-7

[52] Maisnier-Patin S, Roth JR, Fredriksson A, Nyström T, Berg OG, et al (2005) Genomic buffering mitigates the effects of deleterious mutations in bacteria. Nat Genet 37: 1376–1379. 16273106 10.1038/ng1676

[53] Hong JS, Ames BN (1971) Localized mutagenesis of any specific small region of the bacterial chromosome. Proc Natl Acad Sci U S A 68: 3158–3162. 4943557 10.1073/pnas.68.12.3158 PMC389612

[54] Saka K, Tadenuma M, Nakade S, Tanaka N, Sugawara H, et al (2005) A complete set of Escherichia coli open reading frames in mobile plasmids facilitating genetic studies. DNA Res 12: 63–68. 16106753 10.1093/dnares/12.1.63

[55] Emilsson V, Kurland CG (1990) Growth rate dependence of transfer RNA abundance in Escherichia coli. EMBO J 9: 4359–4366. 2265611 10.1002/j.1460-2075.1990.tb07885.x PMC552224

[56] Gehrke CW, Kuo KC, McCune RA, Gerhardt KO, Agris PF (1982) Quantitative enzymatic hydrolysis of tRNAs: reversed-phase high-performance liquid chromatography of tRNA nucleosides. Journal of Chromatography 230: 297–308. 7050138

[57] Gehrke CW, Kuo KC (1990) Ribonucleoside analysis by reversed-phase high performance liquid chromatography. In: Gehrke CW, Kuo KCT, editors. Chromatography and modification of nucleosides. Part A. Analytical methods for major and modified nucleosides. J Chromatography Library. Amsterdam: Elsevier. A3–A71.

[58] Herr AJ, Nelson CC, Wills NM, Gesteland RF, Atkins JF (2001) Analysis of the roles of tRNA structure, ribosomal protein L9, and the bacteriophage T4 gene 60 bypassing signals during ribosome slippage on mRNA. J Mol Biol 309: 1029–1048. 11399077 10.1006/jmbi.2001.4717

[59] Hansen TM, Baranov PV, Ivanov IP, Gesteland RF, Atkins JF (2003) Maintenance of the correct open reading frame by the ribosome. EMBO Rep 4: 499–504. 12717454 10.1038/sj.embor.embor825 PMC1319180

[60] Zahn K (1996) Overexpression of an mRNA dependent on rare codons inhibits protein synthesis and cell growth. J Bacteriol 178: 2926–2933. 8631683 10.1128/jb.178.10.2926-2933.1996 PMC178030

[61] Gurvich OL, Baranov PV, Gesteland RF, Atkins JF (2005) Expression Levels Influence Ribosomal Frameshifting at the Tandem Rare Arginine Codons AGG_AGG and AGA_AGA in Escherichia coli. J Bacteriol 187: 4023–4032. 15937165 10.1128/JB.187.12.4023-4032.2005 PMC1151738

[62] Varshney U, Lee CP, RajBhandary UL (1991) Direct analysis of aminoacylation levels of tRNAs in vivo. Application to studying recognition of *Escherichia coli* initiator tRNA mutants by glutaminyl-tRNA synthetase. J Biol Chem 266: 24712–24718. 1761566

[63] Perona JJ, Rould MA, Steitz TA (1993) Structural basis for transfer RNA aminoacylation by Escherichia coli glutaminyl-tRNA synthetase. Biochemistry 32: 8758–8771. 8364025 10.1021/bi00085a006

[64] Moukadiri I, Prado S, Piera J, Velazquez-Campoy A, Björk GR, et al (2009) Evolutionarily conserved proteins MnmE and GidA catalyze the formation of two methyluridine derivatives at tRNA wobble positions. Nucleic Acids Res 37: 7177–7193. 19767610 10.1093/nar/gkp762 PMC2790889

[65] Hagervall TG, Edmonds CG, McCloskey JA, Björk GR (1987) Transfer RNA(5-methylaminomethyl-2-thiouridine)-methyltransferase from *Escherichia coli* K-12 has two enzymatic activities. J Biol Chem 262: 8488–8495. 3298234

[66] Nilsson K, Lundgren HK, Hagervall TG, Björk GR (2002) The Cysteine Desulfurase IscS Is Required for Synthesis of All Five Thiolated Nucleosides Present in tRNA from Salmonella enterica Serovar Typhimurium. J Bacteriol 184: 6830–6835. 12446633 10.1128/JB.184.24.6830-6835.2002 PMC135462

[67] Lauhon CT (2002) Requirement for IscS in Biosynthesis of All Thionucleosides in Escherichia coli. J Bacteriol 184: 6820–6829. 12446632 10.1128/JB.184.24.6820-6829.2002 PMC135461

[68] Ikeuchi Y, Shigi N, Kato J, Nishimura A, Suzuki T (2006) Mechanistic Insights into Sulfur Relay by Multiple Sulfur Mediators Involved in Thiouridine Biosynthesis at tRNA Wobble Positions. Mol Cell 21: 97–108. 16387657 10.1016/j.molcel.2005.11.001

[69] Chen P, Crain PF, Näsvall SJ, Pomerantz SC, Björk GR (2005) A “gain of function” mutation in a protein mediates production of novel modified nucleosides. EMBO J 24: 1842–1851. 15861125 10.1038/sj.emboj.7600666 PMC1142597

[70] Jäger G, Nilsson K, Björk GR (2010) Reading frame maintenance and how a tRNA selenation enzyme obtains a possible tRNA geranyltranferase activity. In: Weil T, Santos M, editors. 23rd tRNA workshop. From the origin of life to biomedicine. Universidade de Aveiro. 153.

[71] Li JN, Esberg B, Curran JF, Björk GR (1997) Three modified nucleosides present in the anticodon stem and loop influence the in vivo aa-tRNA selection in a tRNA-dependent manner. J Mol Biol 271: 209–221. 9268653 10.1006/jmbi.1997.1176

[72] Björk GR, Hagervall TG (2005) In:Böck A, Curtiss III R, Kaper JB, Neidhardt FC, Nyström T et al., editors. EcoSal - *Escherichia coli* and *Salmonella*. Cellular and Molecular Biology. Washington DC.: ASM Press.

[73] Näsvall SJ, Chen P, Björk GR (2004) The modified wobble nucleoside uridine-5-oxyacetic acid in tRNA^Pro^ _cmo5UGG_ promotes reading of all four proline codons in vivo. RNA 10: 1662–1673. 15383682 10.1261/rna.7106404 PMC1370651

[74] Sroga GE, Nemoto F, Kuchino Y, Björk GR (1992) Insertion (*sufB*) in the anticodon loop or base substitution (*sufC*) in the anticodon stem of tRNA^Pro^ _2_ from *Salmonella typhimurium* induces suppression of frameshift mutations. Nucleic Acids Res 20: 3463–3469. 1630916 10.1093/nar/20.13.3463 PMC312503

[75] Chen P, Qian Q, Zhang S, Isaksson LA, Björk GR (2002) A cytosolic tRNA with an unmodified adenosine in the wobble position reads a codon ending with the non-complementary nucleoside cytidine. J Mol Biol 317: 481–492. 11955004 10.1006/jmbi.2002.5435

[76] Li JN, Björk GR (1999) Structural alterations of the tRNA(m^1^G37)methyltransferase from *Salmonella typhimurium* affect tRNA substrate specificity. RNA 5: 395–408. 10094308 10.1017/s1355838299980834 PMC1369768

[77] Dumelin CE, Chen Y, Leconte AM, Chen YG, Liu DR (2012) Discovery and biological characterization of geranylated RNA in bacteria. Nat Chem Biol 8: 913–919. 22983156 10.1038/nchembio.1070 PMC3494293

[78] Riddle DL, Roth JR (1972) Frameshift suppressors. II. Genetic mapping and dominance studies. J Mol Biol 66: 483–493. 4556576 10.1016/0022-2836(72)90428-7

[79] Dong HJ, Nilsson L, Kurland CG (1996) Co-variation of tRNA abundance and codon usage in Escherichia coli at different growth rates. J Mol Biol 260: 649–663. 8709146 10.1006/jmbi.1996.0428

[80] Jenner LB, Demeshkina N, Yusupova G, Yusupov M (2010) Structural aspects of messenger RNA reading frame maintenance by the ribosome. Nat Struct Mol Biol 17: 555–560. 20400952 10.1038/nsmb.1790

[81] Melnikov S, Ben-Shem A, Garreau de Loubresse N, Jenner L, Yusupova G, et al (2012) One core, two shells: bacterial and eukaryotic ribosomes. Nat Struct Mol Biol 19: 560–567. 22664983 10.1038/nsmb.2313

[82] Rappleye CA, Roth JR (1997) A Tn10 derivative (T-POP) for isolation of insertions with conditional (tetracycline-dependent) phenotypes. J Bacteriol 179: 5827–5834. 9294441 10.1128/jb.179.18.5827-5834.1997 PMC179473

[83] Anderson RP, Miller CG, Roth JR (1976) Tandem duplications of the histidine operon observed following generalized transduction in Salmonella typhimurium. J Mol Biol 105: 201–218. 787532 10.1016/0022-2836(76)90107-8

[84] Leipuviene R, Björk GR (2007) Alterations in the two globular domains or in the connecting {alpha}-helix of bacterial ribosomal protein L9 induces +1 frameshifts. J Bacteriol 189: 7024–7031. 17660285 10.1128/JB.00710-07 PMC2045208

[85] Esberg B, Leung HC, Tsui HC, Björk GR, Winkler ME (1999) Identification of the *miaB* gene, involved in methylthiolation of isopentenylated A37 derivatives in the tRNA of *Salmonella typhimurium* and *Escherichia coli* . J Bacteriol 181: 7256–7265. 10572129 10.1128/jb.181.23.7256-7265.1999 PMC103688

[86] Márquez V, Wilson DN, Tate WP, Triana-Alonso F, Nierhaus KH (2004) Maintaining the ribosomal reading frame: the influence of the E site during translational regulation of release factor 2. Cell 118: 45–55. 15242643 10.1016/j.cell.2004.06.012

[87] Sanders CL, Curran JF (2007) Genetic analysis of the E site during RF2 programmed frameshifting. RNA 13: 1483–1491. 17660276 10.1261/rna.638707 PMC1950767

[88] Liao PY, Gupta P, Petrov AN, Dinman JD, Lee KH (2008) Nucleic Acids Res, in press. 10.1093/nar/gkn100 PMC2377451 18344525

[89] Wolfe MD, Ahmed F, Lacourciere GM, Lauhon CT, Stadtman TC, et al (2004) Functional diversity of the rhodanese homology domain: the *Escherichia coli ybbB* gene encodes a selenophosphate-dependent tRNA 2-selenouridine synthase. J Biol Chem 279: 1801–1809. 14594807 10.1074/jbc.M310442200

